# From environment to organs: individual and combined effects of MPs and PFAS on urinary system health

**DOI:** 10.3389/fphys.2026.1782952

**Published:** 2026-04-07

**Authors:** Dazheng Li, Tianyi Wang, Wenxiu Zhang, Meng Li, Wenzhi Du, Jianning Wang

**Affiliations:** 1 Department of Urology, The First Affiliated Hospital of Shandong First Medical University and Shandong Provincial Qianfoshan Hospital, Jinan, China; 2 Beijing Institute of Heart Lung and Blood Vessel Disease, Beijing Anzhen Hospital, Capital Medical University, Beijing, China; 3 Department of Pediatrics, Shandong Provincial Maternal and Child Health Care Hospital Affiliated to Qingdao University, Jinan, China; 4 Shandong Provincial Hospital, Shandong First Medical University, Jinan, China

**Keywords:** co-exposure, health, microplastics, per- and polyfluoroalkyl substances, urogenital toxicity

## Abstract

As two classes of persistent environmental pollutants, microplastics (MPs) and per- and polyfluoroalkyl substances (PFAS) accumulate in the human urogenital system, posing a significant health threat. To elucidate their toxic mechanisms and inform disease prevention, this review systematically examines their individual and combined toxic effects on the urogenital system, with a focus on the kidneys. Evidence indicates that exposure to MPs induces renal injury primarily by triggering oxidative stress, programmed cell death, and disrupting the gut-kidney axis, with toxicity demonstrating size and carrier dependence. As endocrine-disrupting chemicals, PFAS disrupt metabolic and hormonal homeostasis via pathways such as PPAR activation and can induce epigenetic alterations, leading to metabolic disorders, inflammation, and potential carcinogenic risks. Exposure in real environments often occurs as mixtures of pollutants. However, studies on their combined toxicity remain limited and inconsistent. Existing evidence suggests that MPs can act as carriers for PFAS, but their “carrier effect” does not necessarily directly translate to enhanced biological toxicity. Combined exposure may trigger distinct effects including synergistic, additive, or even antagonistic effects through complex mechanisms such as the modulation of bioavailability and the synergistic amplification of cellular stress, with the specific pattern highly dependent on exposure characteristics and the biological system. Overall, research in this field is still in the early stage, and the interaction mechanisms and health risks of combined exposure urgently require systematic elucidation. Future research urgently needs to integrate multidisciplinary approaches from environmental science, toxicology, and epidemiology. Efforts should focus on developing precise internal exposure characterization techniques, dissecting the toxicological mechanisms of interactions in multi-scale models, and conducting systematic population cohort studies. This is essential for advancing the risk assessment paradigm from single-pollutant evaluation to combined-exposure assessment and ultimately formulating effective public health protection strategies.

## 1 Introduction

Common persistent environmental pollutants include microplastics (MPs) and per- and polyfluoroalkyl substances (PFAS). Owing to their exceptional durability and versatility, plastic products have become ubiquitous in modern life; however, their large-scale production, utilization, and disposal pose significant global environmental challenges. The term “microplastics” originated from the work of Thompson et al. in a 2004 publication in *Science* (Thompson et al., 2004). Defined as plastic particles measuring less than 5 mm, MPs are categorized into two subgroups: primary microplastics and secondary microplastics (Zhang et al., 2020; La Porta et al., 2023). A study published in *The Lancet* highlights that plastic pollution is escalating rapidly, inflicting health-related economic losses of at least 1.5 trillion U.S. dollars annually. In the absence of effective interventions, this figure is projected to reach 1.2 billion tons by 2060 (Landrigan et al., 2025). Another archetypal pollutant, PFAS, comprises a class of synthetic compounds characterized by their extraordinary chemical stability and persistence (Quan et al., 2025). These substances are extensively employed in industrial processes and consumer products. As research advances, scientists have consistently detected pollutants such as MPs in organs, including the liver and lungs, as well as in association with various pathological conditions (Horvatits et al., 2022; Golden-Mason et al., 2025; He et al., 2025; Dou et al., 2026). Recent investigations have demonstrated that MPs can traverse the placental barrier (Mathew et al., 2025), suggesting a high probability of these pollutants being present in fetal tissues. Notably, accumulating evidence has verified that these two classes of plastic pollutants induce varying degrees of adverse effects on human health (Guo et al., 2025; Jiang et al., 2025; Wei et al., 2025; Wu et al., 2025).

As the primary excretory and metabolic organs of the human body, the urinary system is directly responsible for filtering toxic substances from the bloodstream to form urine. It is continuously and directly exposed to environmental pollutants transported via the blood, thereby emerging as a key target of toxic effects. Accumulating evidence confirms the detection of these pollutants within the human urinary system. Massardo et al. first detected MPs in kidney specimens and urine, confirming their ability to reach the kidneys and be excreted in the urine (Massardo et al., 2024). Recent reviews have demonstrated that MPs are present in 54% of urine samples, 70% of kidney tissues, and 68% of bladder cancer cases (O’Callaghan et al., 2025), providing preliminary direct evidence for the potential association between MPs and urinary system diseases. Unlike other pollutants, PFAS are proteinophilic rather than lipophilic (Gong et al., 2025). Given the kidneys’ high blood flow, large endothelial surface area, and abundant tissue protein content, they serve as the preferential target organ for PFAS accumulation. Humans cannot metabolize PFAS compounds, such as perfluorooctanoic acid (PFOA), which leads to their long-term retention in the body. A substantial body of research has confirmed that PFAS can increase the risk of renal function impairment, hyperuricemia, and even tumorigenesis through mechanisms including metabolic disruption and abnormal epigenetic regulation (Liu et al., 2023; Niu et al., 2024; Sands et al., 2024; Zhang et al., 2024).

**Figure 1:**
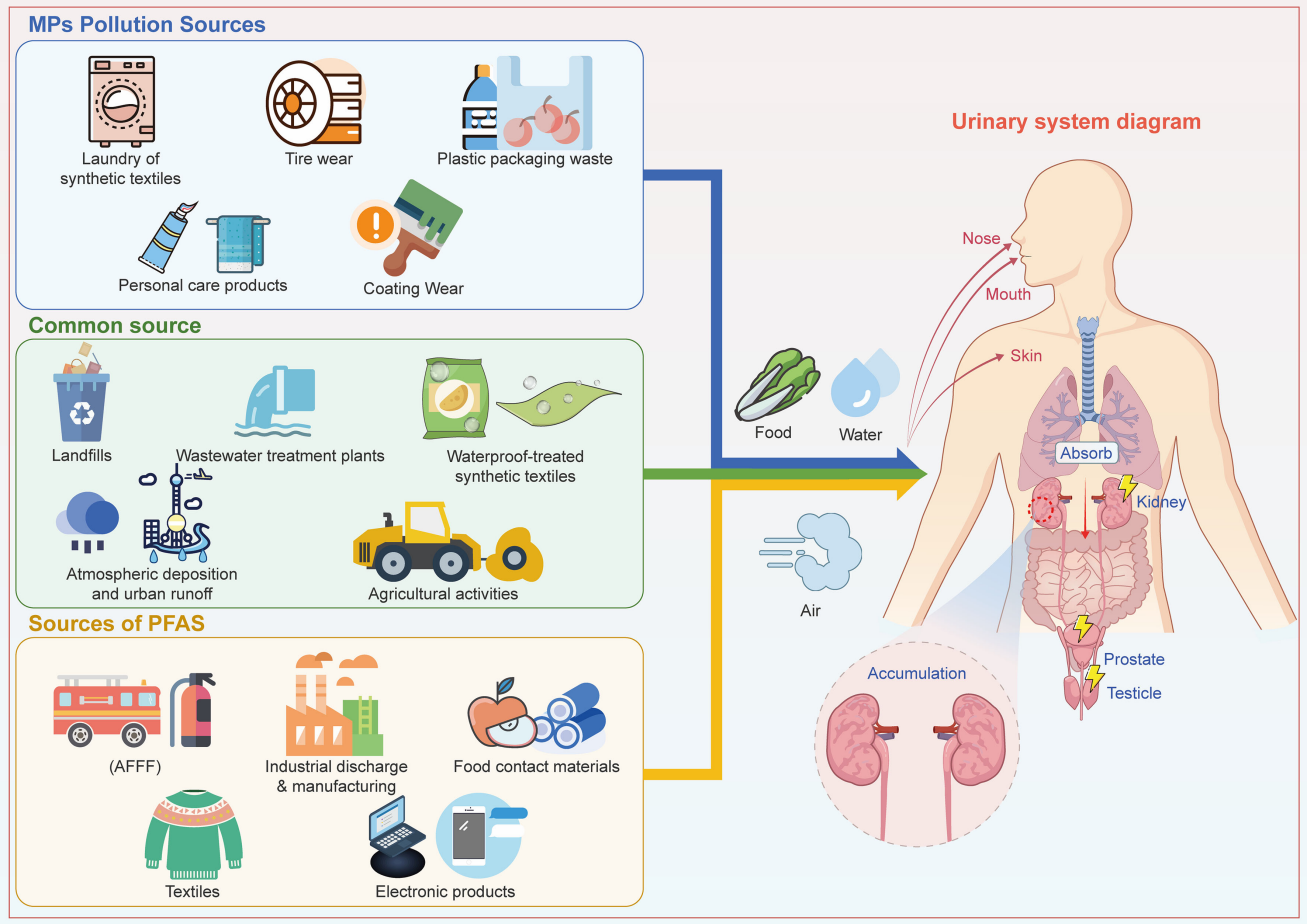
Sources, environmental pathways, and exposure routes of PFAS and MPs leading to urinary system accumulation.

Currently, the individual toxic mechanisms of MPs and PFAS on the urogenital system have been extensively investigated. However, their environmental distributions are not independent. (**Figure 1**) Empirical studies have detected both pollutants concurrently in various media, including estuarine surface waters (Cheng et al., 2021), agricultural watersheds (Meppelink et al., 2025), urban sewer overflows (Butzlaff et al., 2025), and groundwater (Yashir et al., 2025), with co-occurrence also confirmed in biological samples such as sea turtles, dolphins (Navarathna et al., 2023), and Mediterranean mussels (Vega-Herrera et al., 2024). Their co-occurrence arises primarilyfrom shared emission sources and convergence pathways (e.g., wastewater treatment systems, landfill leachate, and urban runoff) that facilitate synchronous release (Prada et al., 2024; Butzlaff et al., 2025; Ma et al., 2025). Additionally, complex physicochemical interactions contribute significantly, including the carrier effect and the function of plastics as secondary release sources for PFAS (Cheng et al., 2021; Salawu et al., 2024; Ma et al., 2025). This indicates that humans face consistent exposure to pollutant mixtures in real-world environments, and the current single-pollutant research paradigm cannot accurately assess the associated combined health risks. Notably, studies on the co-toxicity of these two pollutants to the urogenital system remain extremely scarce. In this context, this review aims to make the first systematic inference and demonstration of the potential mechanisms and risks of their combined toxicity, based on existing single-toxicity data. The goal is to provide a scientific basis for future mechanistic exploration, risk assessment, and the development of health protection strategies.

## 2 Mechanisms of MPs-associated urinary toxicity

### 2.1 Exposure routes and renal target organ sensitivity

MPs, as ubiquitous environmental contaminants, primarily enter the human body through the ingestion of contaminated food, inhalation of airborne particles, exposure via medical procedures or implants, and transplacental transfer (Amobonye et al., 2021; Ragusa et al., 2022; Passos et al., 2023; Casella et al., 2025; Yakovenko et al., 2025). (**Figure 1**) Their ability to reach the urogenital system is highly dependent on particle size. The potentially hazardous size range spans from micrometers down to nanometers. In human samples, micro-Raman spectroscopy has identified plastic fragments measuring 1-29 μm, 3-13 μm, and 2-6 μm in kidney tissue, urine, and semen, respectively (Montano et al., 2023; Massardo et al., 2024). Other studies using laser direct infrared imaging (LDIR) have widely reported MPs of 20-100 μm in testes and semen (Zhao et al., 2023; Guo et al., 2025). Given the low detection sensitivity of this method for particles below 20 μm, which may underestimate the abundance of smaller MPs. In contrast, animal studies demonstrate that nanoplastics (NPs) (e.g., 50 nm polystyrene) can accumulate in murine kidneys and testes following oral exposure (Meng et al., 2022b; Lv et al., 2025), indicating that nanoscale particles, which possess greater tissue penetration potential, may also access the human urogenital system, though direct evidence remains limited.

In summary, MPs directly detected in the human urogenital system are predominantly micrometer-sized, while NPs constitute a potential risk. The reported size distribution is heavily influenced by the sensitivity and selection bias of the analytical methods employed. Building upon the evidence of MP entry and distribution, it is crucial to examine their biological effects within the urogenital system.

**Figure 2:**
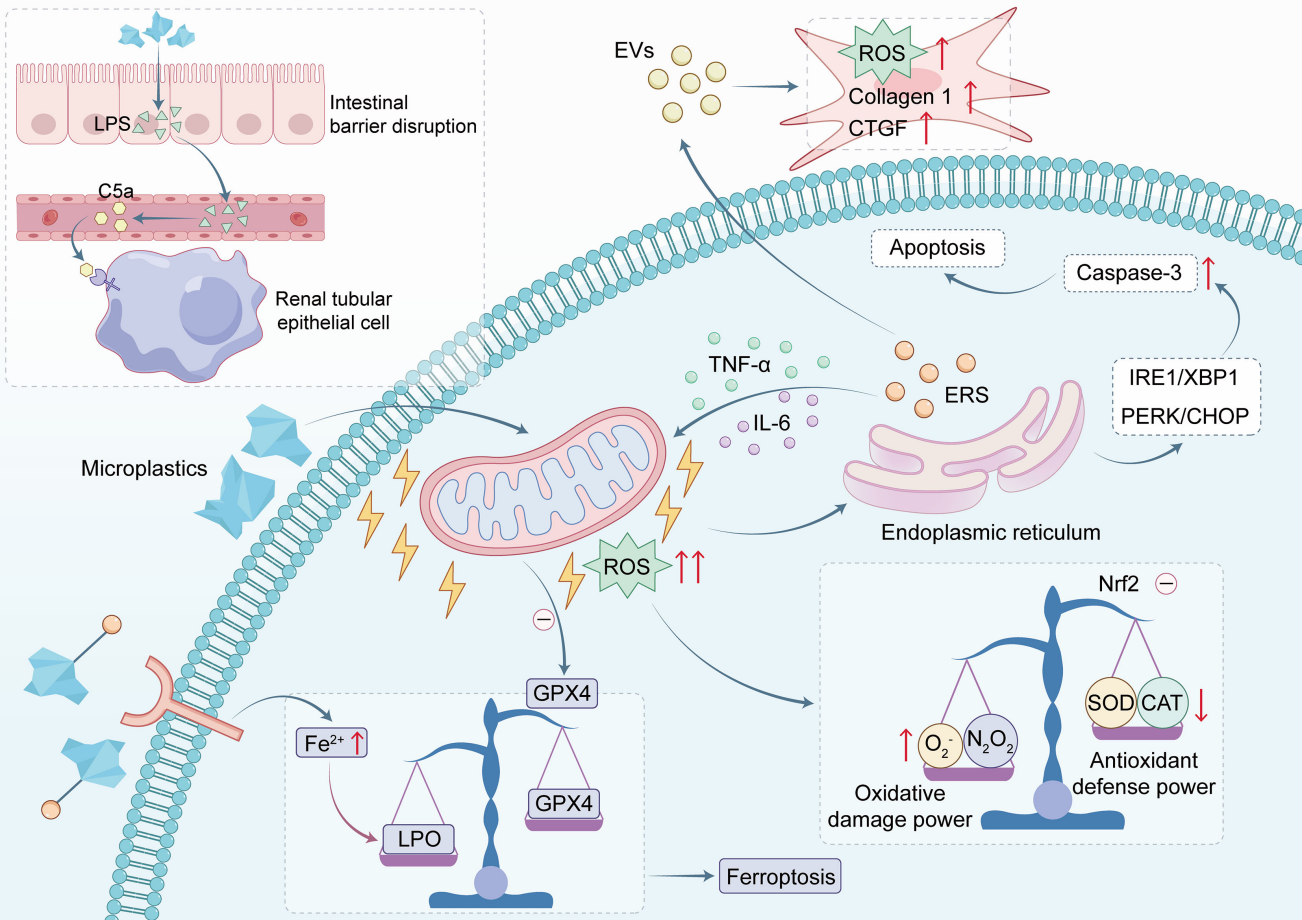
Core mechanistic pathways of MPs toxicity in the urinary system.

As the primary organs responsible for blood filtration and waste excretion, the kidneys represent a critical target for the accumulation and toxicity of MP. Renal cells appear to exhibit heightened sensitivity to MPs. Studies indicate that under comparable exposure conditions, renal cells can experience higher mortality and more significant declines in cellular metabolic activity than other cell types (Goodman et al., 2022; Li et al., 2023). Similarly, Chen L et al. reported that renal cells exhibit greater sensitivity to plastic exposure compared to other cell lines (Chen et al., 2025). Of particular clinical concern are patients with renal impairment, especially those on long-term dialysis. Due to their compromised excretory function and reliance on dialysis solutions and PVC-based medical devices, these patients may face elevated body burdens of MPs and an increased risk of toxicity (Posnack, 2014). Research by Wang et al. suggests a potential association between MP exposure and the onset of hypertension after kidney transplantation (Wang et al., 2025). However, the underlying mechanisms remain to be elucidated. The nephrotoxicity induced by MPs is a progressive process, involving interconnected molecular, cellular, and organ-level disturbances. (**Figure 2**) (**Table 1**).

**Table 1:** Several studies on MPs-induced urogenital toxicity.

Pollutant type/size	Experimental model	Experimental subject	Exposure conditions(concentration & duration)	Key effects	Main mechanisms	Ref.
Polystyrene Microplastics (1μm)	*In vitro* cellular model	HEK 293	Various concentrations, up to 100 μg/mL; 24h, 48h, 72h	• Cell proliferatio↓ Cell viability ↔; • Intracellular ROS↑; • Gene expression: SOD2, CAT, GAPDH↓;	Induction of oxidative stress coupled with the suppression of endogenous antioxidative and metabolic defenses.	(Goodman et al., 2022)
Polystyrene Microplastics (1,000 nm)	*In vivo* animal model	Juvenile rats	2.0 mg/kg/d; 28 days	• Renal function markers (BUN, CRE)↑; • Histopathological damage • Oxidative stress, ER stress, inflammation↑; • Apoptosis↑;	Oxidative stress-ER stress-apoptosis axis: PS-MPs trigger oxidative stress→activate ER stress→induce inflammation and apoptosis via Caspase-12.	(Wang et al., 2022)
Polystyrene Microplastics (2μm)	Combined *in vitro* and *in vivo* models	HK-2 cells; Male C57BL/6 mice	• *In vitro*: 0.025-0.8 mg/mL for 6h to 3d. • *In vivo*: 0.2 or 0.4 mg/day for 4 or 8 weeks.	• Mitochondrial ROS↑, pro-apoptotic Bad↑; • ER stress, inflammation, autophagy activation↑;	Mitochondrial ROS as the key initiator: PS-MPs induce mitochondrial ROS burst→upstream trigger for ER stress, inflammatory response, and autophagic flux.	(Wang et al., 2021)
Polylactic Acid Microplastic (1 μm)	Combined *in vitro* and *in vivo* models	TM 4, GC-2, and Male ICR mice	• *In vivo*: 10, 100, 1000 mg/kg/d for 8 weeks. • *In vitro*: 0, 20, 100, 500μg/mL for 24h or 48h.	• Blood-testis barrier integrity↓; • Sperm quality↓ (concentration, motility↓; deformity↑) • Sperm mitochondrial structure/function↓; • Spermatogenesis-related mRNA↓;	PLA-derived nanoparticles penetrate the blood-testis barrier→localize to and disrupt the mitochondrial sheath of sperm→impair mitochondrial function and related gene expression.	(Zhao et al., 2025)
Polystyrene Microplastics (100 nm, 1μm) and Cadmium	*In vivo* animal model	Kunming mice	• Cd: 5 mg/kg/d; • PS: 100 mg/kg/d for 25 days.	• Size-dependent toxicity: 100 nm PS > 1 μm PS; • Combined effect: Cd + PS > single exposure; • Apoptotic proteins (Bax/Bcl-2 ratio, p53, Caspase-3)↑;	Carrier effect and synergistic apoptosis: Nano-PS acts as a carrier for Cd, enhancing its bioavailability and jointly activating the mitochondrial apoptotic pathway (p53/Bax/Caspase-3).	(Zhang et al., 2024)
UV-Aged Polystyrene Nanoplastics (124 nm)	Combined *in vitro* and *in vivo* models	HK-2 cells; Male ICR mice	• *In vivo*: 50 mg/kg/d for 28 days. • *In vitro*: 0-1000 μg/mL for 12h.	• Renal function markers (BUN, CRE)↑; • Mitochondrial damage↑; • Ferroptosis markers (Fe^2+^, ROS↑; GSH, GPX4↓);	Aging-enhanced transferrin hijacking induces ferroptosis: UV-aging increases aPS-NP bioactivity and transferrin (TF) adsorption→aPS-TF complexes enhance cellular uptake via TF-receptor→causes iron overload and GPX4 depletion, triggering ferroptosis.	(He et al., 2024)
Polystyrene Microplastics (1μm)	*In vitro* cellular model	LNCaP and primary cultured human prostate cancer cells	Focus on low-dose (0.1μg/mL); compared with higher doses for 48h.	• Low-dose specific effect: Promoted cell proliferation↑ • Induced oxidative stress & inflammation (ROS↑) • Inhibited ferroptosis (GPX4↑, ACSL4↓)	GPX4-mediated ferroptosis inhibition promotes proliferation: Low-dose PS-MPs→GPX4/ACSL4 upregulation→Ferroptosis inhibition→Reduced cell death→Enhanced prostate cancer cell proliferation.	(Li et al., 2025)
Polystyrene Microplastics (2μm)	Combined *in vitro* and *in vivo* models	Male C57BL/6 mice; HK-2 cells	• *In vivo*: 0.5 or 2 mg/kg/d for 8 weeks. • *In vitro*: 10–300 mg/L for 24h.	• Renal dysfunction markers (BUN, urinary albumin)↑; • Renal pathology (glomerulosclerosis, fibrosis)↑; • Gut barrier disruption (permeability, plasma LPS)↑; • Complement activation (urinary C5a, renal C5aR)↑;	Gut-renal axis via complement: Oral PS-MPs impair the intestinal barrier→increased systemic LPS triggers complement-mediated renal inflammation (via C5a/C5aR), leading to kidney injury.	(Liang et al., 2024)

↑, increase; ↓, decrease; ↔, no significant change.

### 2.2 Initiation and activation: oxidative stress-driven pathways

The high metabolic activity and oxygen demand of the kidneys predispose them to oxidative stress (Moriya et al., 2025; Mushuo et al., 2026). Oxidative stress induced by MPs constitutes a primary initiating event in MP-induced nephrotoxicity. Following their uptake by renal tubular epithelial cells, MPs can cause organelle dysfunction, particularly in mitochondria, leading to the excessive generation of reactive oxygen species (ROS). This has been evidenced in various models, including oral exposure of young mice to 1000 nm polystyrene microspheres (2.0 mg/kg/day; smooth, spherical particles), and *in vitro* treatments with 1μm or 2 μm polystyrene microspheres on human renal cells (e.g., HEK 293, HK-2 cells) (Wang et al., 2021; Goodman et al., 2022; Wang et al., 2022). Moreover, treatment with PS-MPshas been shown to impair the Nrf2 signaling pathway in relevant model (Huang et al., 2025), which orchestrates the core antioxidant response. This impairment disrupts the oxidant-antioxidant equilibrium by diminishing the activity of key enzymes, including SOD and CAT (Ahmed et al., 2022; Goodman et al., 2022). The resultant oxidative imbalance triggers a cascade of damage, including lipid peroxidation, protein denaturation, and DNA injury (Zou et al., 2022; Qiu et al., 2023). Related studies on reproductive toxicity corroborate this mechanistic pathway. Zhao et al. demonstrated that in mice, chronic oral exposure to PLA microplastics (at doses up to 1000 mg/kg/day for 56 days) can cross the blood-testis barrier, accumulate in the testes, and impair spermatogenesis. This effect was mediated through mechanisms including blood-testis barrier disruption, mitochondrial dysfunction, and oxidative stress, ultimately leading to reduced sperm concentration, impaired motility, and hormonal disorders in mice (Zhao et al., 2025). Similarly, research by Jeon et al. and Wei et al. reported that polystyrene nanoparticles induce a surge of ROS, which alters the expression of actin-binding proteins, compromises the blood-testis barrier integrity, and causes sperm dysfunction (Wei et al., 2021; Jeon et al., 2024).

Oxidative stress acts not only as the initiating event but also as the central driver of downstream damaging cascades in microplastic-induced nephrotoxicity. On one hand, excessive ROS induces endoplasmic reticulum stress (ERS), activating signaling pathways such as IRE1/XBP1 and PERK/CHOP. This activation disrupts the balance between pro-apoptotic and anti-apoptotic proteins, upregulates Caspase-3, and consequently initiates the apoptotic program (Wang et al., 2021; Wang et al., 2022; Zou et al., 2022; Li et al., 2023; Zhang et al., 2024). On the other hand, the ROS burst activates the canonical NF-κB inflammatory pathway by promoting IκBα phosphorylation and degradation (Wang et al., 2021; Liang et al., 2024), while concurrently facilitating NLRP3 inflammasome activation (Feng et al., 2024; Kuang et al., 2024). These processes collectively promote the release of numerous pro-inflammatory cytokines, such as TNF-α, IL-1β, and IL-6, and inhibit the TGF-β/Smad signaling pathway. The resultant effects include a robust inflammatory response, inhibition of the epithelial-mesenchymal transition (EMT), exacerbation of tissue damage and fibrosis, and the induction of pyroptosis. Critically, these pathways form a self-amplifying, vicious positive feedback cycle (Meng et al., 2022a; Feng et al., 2024). Inflammatory cytokines released via ERS and NLRP3 activation can feed back to exacerbate mitochondrial dysfunction, further driving ROS production. Conversely, persistently elevated ROS levels positively regulate both ERS and inflammatory signaling. This sustained, intensifying cycle of damage promotes more severe programmed cell death and tissue fibrosis, thereby laying the pathological foundation for the progressive deterioration of renal function (Xiong et al., 2023; Wang et al., 2024).

Beyond intracellular self-amplification, damaging signals can also propagate intercellularly via specific pathways, ultimately contributing to tissue-level lesions. Harmful communication mediated by extracellular vesicles (EVs) is a key link in the development of renal fibrosis. Studies indicate that MP-inducedendoplasmic reticulum stress in renal tubular epithelial cells significantly enhances EV release. *In vitro* and *in vivo* experiments by Wang et al., using polystyrene microspheres (2.1~2.2 μm) at concentrations of 0.4/0.8 mg/mL in cells and oral doses of 0.2/0.4 mg/day in mice, have demonstrated that polystyrene promotes the expression of the EV marker CD63 and induces oxidative stress. Upon internalization by recipient fibroblasts, these EVs derived from stressed tubular cells trigger ROS production and upregulate the expression of fibrotic markers in the fibroblasts, thereby directly driving the progression of renal fibrosis (Wang et al., 2024).

### 2.3 Key execution mechanisms: programmed cell death

Programmed cell death pathways are activated in response to oxidative stress and inflammation. Ferroptosis, a recently characterized form of iron-dependent regulated cell death, plays a significant role in this context. Professor He elucidated the core mechanism of microplastic-induced ferroptosis. Their findings indicate that polystyrene nanoparticles (PS NPs and their aged counterparts, aPS NPs)can bind to transferrin (TF) in the bloodstream, forming complexes that are internalized via the transferrin receptor 1 (Tfr1), thereby inducing intracellular iron overload. The accumulated Fe²^+^ catalyzes the excessive production of lipid peroxides (LPO). Concurrently, MP-generated ROS inhibits the activity of the key antioxidant enzyme GPX4. This dual action prevents the clearance of LPO, ultimately leading to the disintegration of cellular membranes and ferroptosis, as demonstrated in both HK-2 cells (exposed up to 1000 μg/mL) and in mice following oral exposure (50 mg/kg/day for 28 days) (Cheng et al., 2024; He et al., 2024). Building upon this mechanism, a study by Hong et al. further established that ferritinophagy-mediated ferroptosis in renal tubular epithelial cells is a central event in polystyrene-induced renal fibrosis, with its upstream regulation driven by oxidative stress and inflammation. Notably, the ferroptosis inhibitor Fer-1 can effectively mitigate such damage, highlighting a potential therapeutic target (Hong et al., 2024). However, Li et al. reported a potentially divergent effect in prostate cancer cells. Their *in vitro* study on LNCaP cells showed that low-dose polystyrene (1 μm PS-MPs at 0.1 μg/mL for 48 hours) did not induce ferroptosis. Instead, they upregulated the expression of GPX4 and ACSL4, which may help cancer cells evade ferroptosis. Furthermore, low-dose polystyrene promoted cancer cell proliferation by inducing moderate oxidative stress and altering the secretion of inflammatory cytokines to facilitate immune evasion, collectively fostering tumor progression (Li et al., 2025).

### 2.4 Indirect mechanisms and cross-organ communication: the role of the gut-kidney axis

Beyond directly damaging the kidneys, MPs can also induce renal injury through the gut-kidney axis. Certain MPs accumulate in the intestines, disrupting intestinal barrier integrity, inducing dysbiosis and local inflammation. This cascade promotes the translocation of lipopolysaccharide (LPS), a potent bacterial endotoxin, into the systemic circulation (Ghosh et al., 2020). In the bloodstream, LPS activates the complement system, leading to the generation of substantial amounts of C5a. Subsequently, C5a travels to the kidneys, binds to C5a receptors on renal tubular epithelial cells, and recruits damaging factors, such as TGF-β, thereby indirectly triggering glomerular injury and interstitial inflammation, which ultimately accelerates the progression of renal fibrosis. Crucial evidence for this pathway was provided by Liang et al. Their study demonstrated that in mice that, following 8-week oral exposure to 2 μm polystyrene microspheres (at 0.5 or 2 mg/kg/day), repair of the intestinal barrier with antibiotics significantly reduced urinary C5a levels and alleviated renal injury (Liang et al., 2024), thereby confirming the mediating role of the gut-kidney axis.

### 2.5 Key determinants: particle size-dependent effects and combined toxicity

The toxic effects of MPs are significantly regulated by their intrinsic physical properties and environmental factors. Research by Lin Z et al. on male reproduction in mice showed that oral exposure to smaller polystyrene particles (25-30 nm and 1-5 μm) caused more severe impairment to sperm motility and uniquely invaded specific testicular cells (Lin et al., 2024). Similarly, Xiong X et al. found that exposure to different sizes of polystyrene particles induced kidney injury through distinct molecular mechanisms: 80 nm particles primarily triggered immune responses, while 0.5 μm and 5 μm particles disrupted circadian rhythm pathways (Xiong et al., 2023). Both investigations, utilizing spherical polystyrene beads, confirm that particle size is a critical determinant of biological impact, even for simplified model particles. Meng X et al.’s finding further validated this observation and further showed that the relationship is not a simple linear one. Specifically, they found that particles of approximately 600 nm tend to accumulate in regions such as the glomerular mesangium, causing local obstruction and ROS outburst. Consequently, these particles may exert more pronounced damaging effects compared to smaller or larger particles (Meng et al., 2022b).

Importantly, compared to the individual toxic effects of MPs, the synergistic toxicity induced by their co-exposure with other environmental pollutants is more pronounced. In the environment, MPs can act as “Trojan horses”: their large specific surface area allows them to effectively adsorb pollutants, such as heavy metals and organic compounds, forming complexes that exert enhanced combined toxicity (Dissanayake et al., 2022). For instance, combined exposure to MPs such as 5 μm polystyrene microspheres (at a concentration of 10 mg/L in drinking water)and cadmium can significantly exacerbate oxidative stress, dysregulate mitophagy, and increase the expression of proteins related to fibrosis (Zou et al., 2022). Moreover, co-exposure to di-(2-ethylhexyl) phthalate (DEHP) can overactivate the CYP450 enzyme system and the NF-κB/NLRP3 pathway, leading to severe inflammation and pyroptosis, the combined effect of which far exceeds the toxicity of either pollutant alone (Li et al., 2024). Similarly, Zhou et al. demonstrated that combined exposure to polystyrene and DEHP induces oxidative stress, activates PPARγ, ultimately leading to lipid metabolism imbalance and testicular injury (Zhou et al., 2025). The toxicity of MPs can also be amplified through interaction with dietary factors. Research shows that even low-dose exposure to MPs, when combined with an unhealthy dietary pattern, can result in significant synergistic harm. For instance, Gao et al. used spherical PS-MPs with a particle size of 1.0 μm, exposing mice via drinking water at an extremely low dose of 25-30 μg/kg body weight per dayfor 12 weeks. They found that this low-dose PS-MP exposure, when combined with a high-fat diet, synergistically triggered severe inflammation and epithelial cell apoptosis in the mouse prostate, thereby impairing fertility (Gao et al., 2024). Similarly, Xu et al. used 1 μm spherical PS-MPsat a concentration of 10 mg/Lin drinking water for 18 weeks. Combined with single-cell RNA sequencing analysis, their study revealed that PS-MPs together with a high-fat diet remodeled the cellular composition and function of the kidney, exacerbating renal injury and the fibrotic microenvironment (Xu et al., 2024). Together, these two studies demonstrate that small-sized, spherical PS-MPs​, when combined with the metabolic stress of a high-fat diet, can produce compounded toxic effects that exceed those of either factor alone. This combined exposure also led to a significant reduction in seminal plasma nutrients such as zinc and citrate, suggesting that MPs may exacerbate toxicity by interfering with organismal metabolism and disrupting the internal homeostasis of the reproductive system.

## 3 Mechanisms of PFAS-associated urinary toxicity

### 3.1 Exposure routes and renal target organ sensitivity

PFAS, as a class of persistent pollutants, can enter the human body through multiple exposure routes. The predominant route is the ingestion of contaminated food and water (Sunderland et al., 2019; Abraham and Monien, 2022; Fillman et al., 2025). Significant additional exposure occurs via inhalation of indoor air and dust, direct contact through medical devices (e.g., catheters, stents), and maternal-fetal transmission through the placenta and breastfeeding (Sunderland et al., 2019; Zheng et al., 2022; Briassoulis et al., 2025). Furthermore, PFAS are ubiquitous in consumer products, including personal care items (e.g., cosmetics, sanitary products) and daily goods (e.g., non-stick cookware, waterproof textiles, firefighting equipment) (Abraham and Monien, 2022; Dewapriya et al., 2023; Céline et al., 2024). These diverse and continuous sources collectively form the basis for PFAS entry and gradual accumulation in humans.

Accumulating evidence confirms the presence of PFAS in the human urogenital system. Studies in the context of male infertility in China reported detection rates of 96.1%-100% for perfluorooctanoic acid (PFOA), perfluorooctanesulfonic acid (PFOS), and 6:2 Cl-PFESA in semen, with median concentrations of 0.229, 0.097, and 0.064 ng/mL, respectively (Pan et al., 2019). Similarly, research on women undergoing assisted reproduction revealed widespread exposure. In an Australian IVF cohort (2006-2011), PFOS, PFOA, perfluorohexanesulfonic acid (PFHxS), perfluorononanoic acid (PFNA), perfluorodecanoic acid (PFDA), perfluoroheptanesulfonic acid (PFHpS), perfluoroundecanoic acid (PFUnDA), and perfluoroheptanoic acid (PFHpA) were detected in most samples, with median concentrations ranging from 0.01 to 4.8 ng/mL (Kim et al., 2020). A large Chinese IVF cohort detected n-PFOS, PFDA, PFHxS, PFNA, and PFUnDA in 729 follicular fluid samples (median: 0.13-1.70 ng/mL). In addition, the detection rates of n-PFOA, PFHpS, and perfluorotridecanoic acid (PFTrDA) all exceeded 90%, with median concentrations of 1.09, 0.04, and 0.07 ng/mL, respectively (Zeng et al., 2023). Direct evidence of organ accumulation comes from a Danish autopsy study. PFHxS, PFOA, PFOS, and PFNA were detected in 94.7%-100% of kidney tissues at concentrations of 0.494, 0.317, 2.174, and 0.147 ng/g, respectively, with specific renal enrichment noted for PFHxS. A high correlation between kidney and blood concentrations supports the use of blood biomonitoring as a proxy for internal burden (Nielsen et al., 2024).

**Figure 3:**
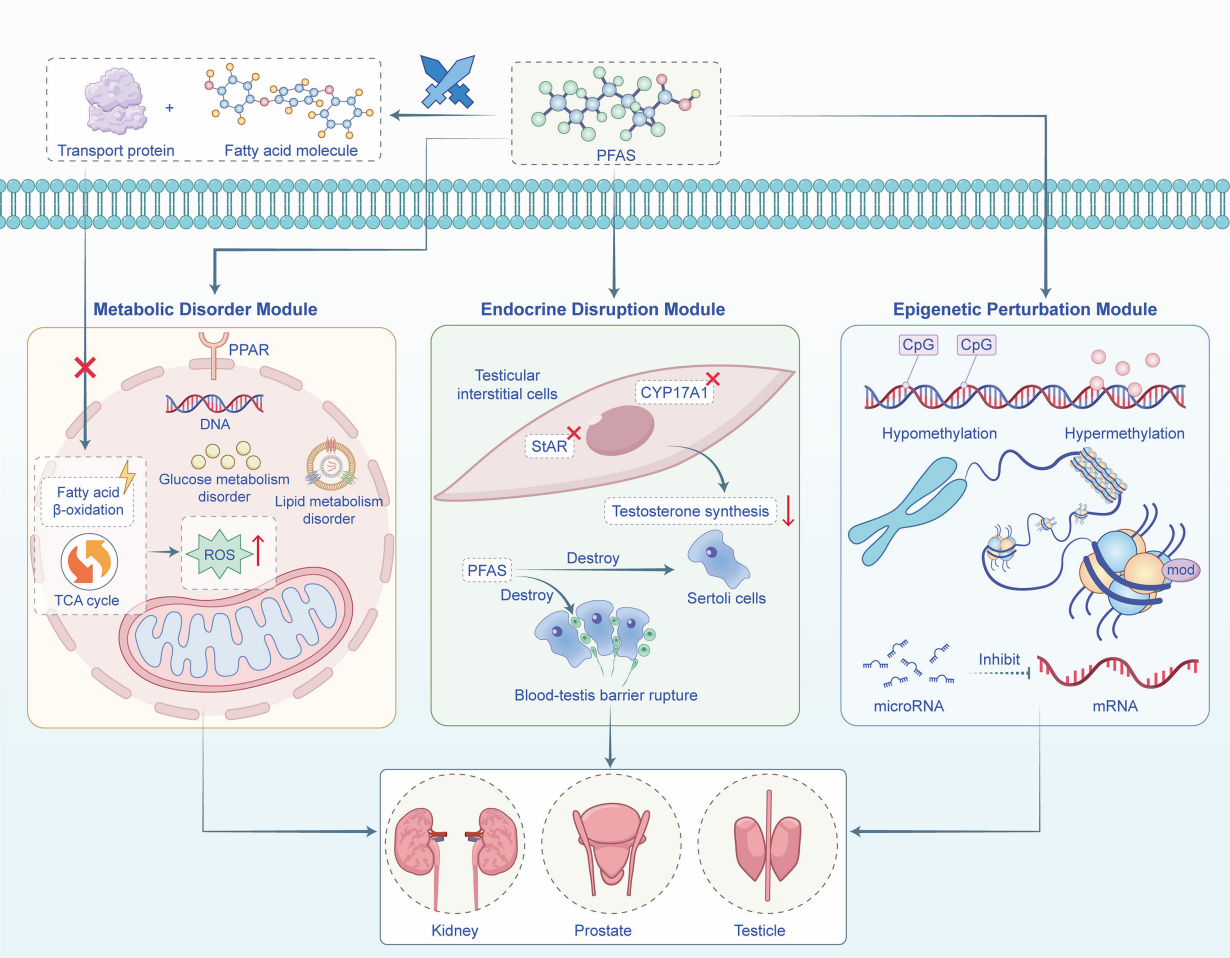
Schematic diagram of the key toxicity mechanisms of PFAS in the urinary system.

Collectively, current data conclusively demonstrate widespread internal exposure and tissue accumulation of PFAS within the human urogenital system. However, publicly available data quantifying PFAS in specific tissues such as the bladder or prostate remain scarce, as biomonitoring still primarily relies on blood and non-invasive matrices (e.g., urine, breast milk). Hence, future studies are needed to deepen and refine our understanding of organ-specific PFAS distribution and its health implications. Based on the aforementioned exposure and accumulation profiles, the toxic effects of PFAS on the urogenital system also manifest through multifaceted and complex mechanisms. (**Figure 3**) (**Table 2**).

**Table 2:** Several studies on PFAS-induced urogenital toxicity.

Pollutant type	Experimental model	Experimental subject	Exposure conditions(concentration & duration)	Key effects	Main mechanisms	Ref.
PFOS and PFBS	Combined *in vitro* and *in vivo* models	Prostate epithelial/cancer cell lines (RWPE-1, RWPE-kRAS); Nude mice bearing RWPE-kRAS	• *In vitro*:10^-5^ to 10^-^¹^0^ M for 1 week. • *In vivo*:10 mg/kg/d for 40 days, with concurrent high-fat diet or control diet	• Prostate cancer cell proliferation↑; • Synergizes with high-fat diet to accelerate prostate tumor growth↑; • Alters cellular metabolism (glycolysis↑, pyruvate↑, acetyl-CoA↑)	Metabolic reprogramming fuels epigenetic dysregulation: PFOS/PFBS induce metabolic reprogramming (enhancing glycolysis and pyruvate metabolism)→increase acetyl-CoA levels→drive histone hyperacetylation and chromatin remodeling→activate oncogenic signaling (e.g., PPARα) to promote tumor growth.	(Imir et al., 2021)
PFOS and HQ-115	Combined *in vitro* and *in vivo* models	TGCT cell lines (2102EP, NT2/D1); Immunodeficient nude mice with xenografts	• *In vitro*: 10 pM – 1 mM for 4 days; • *In vivo*: PFOS: 10 mg/kg/d; HQ-115: 1 or 10 mg/kg/d; 15 days;	• Promotes tumor growth *in vivo*; • Induces epigenetic reprogramming (H3K27me3/Polycomb pathway↓, DNA methylation alterations); • Alters metabolic profiles, enriching pathways in fatty acid homeostasis and steroid biosynthesis;	• PFOS and HQ-115 downregulate the H3K27me3-mediated Polycomb pathway and disrupt DNA methylation. • Act as fatty acid analogs to dysregulate fatty acid homeostasis and steroid synthesis, collectively driving tumor growth.	(Boyd et al., 2024)
PFO4DA	Combined *in vitro* and *in vivo* models	Adult male BALB/c mice; TM3 and TM4 cell lines	• *In vivo*: 2 & 10 mg/kg/d for 28 days. • *In vitro*: 20、50、125 μM for 24h.	• Reproductive function impairment: Sperm count↓, sperm deformity rate increased 2–3 times; • Hormonal disorder: Testosterone level decreased by > 60%, with no change in LH and FSH; • Blood-testis barrier integrity↓; • Expression of steroidogenesis-related genes (StAR and CYP11A1) and proteins↓;	Inhibition of steroidogenesis via PI3K/AKT/mTOR pathway suppression: PFO4DA directly inhibits the PI3K/AKT/mTOR signaling pathway, leading to the downregulation of key steroidogenic enzymes (e.g., StAR, CYP11A1) and ultimately disrupting testosterone synthesis.	(Mutalifu et al., 2025)
PFOS	*In vivo* animal model	Adult male CD-1 mice	5, 10, 20 mg/kg/d for 14 days.	• Gene expression alterations (renal injury markers, key TFs). • Epigenetic reprogramming: global DNA hypomethylation (↓42%), histone demethylases(Kdm1a/Kdm4c)↑; • Fibrosis/metabolism/cancer-related pathways activation;	Global DNA hypomethylation & histone demethylase (Kdm1a/Kdm4c)↑→Transcriptional dysregulation (PPARs, Nrf2, etc.)→Downstream pathway activation (fibrosis, metabolism, cancer).	(Wen et al., 2022)
PFOA	Combined *in vitro* and *in vivo* models	LNCaP, 22RV1 cell lines; BALB/c nude mice with xenografts	• *In vitro*: 25, 50 µM for 24h (part 48h) • *In vivo*: 0.02 mg/kg/d for 30 days.	• PCa cell proliferation, migration, invasion↑; • Autophagy↑(Atg5, Atg7↑; p62↓) • MAPK15 expression↑; • Global m6A methylation↑; FTO demethylase activity↓;	FTO activity↓→m6A↑→MAPK15 expression↑→Autophagy activation→Enhanced PCa proliferation/migration/invasion	(Qian et al., 2025)
PFOA and PFOS	Human cross-sectional study & Computational simulation	Young men (18–35 yr) from a PFAS-contaminated area (n=99) vs. a non-contaminated area (n=50) in Italy	Chronic environmental exposure (residence in contaminated area)	• Abnormal protamine-histone ratio in 72.7% of samples; • Severe and prevalent sperm DNA damage (Grade 3 fragmentation).	PFOA binding to DNA bases & protamine arginine residues→impaired chromatin compaction→loss of DNA protection (especially under oxidative stress)→oxidative damage accumulation→sperm DNA fragmentation	(Musella et al., 2025)
PFNA	*In vivo* animal model	ICR mice	0.5 & 3 mg/kg/d; Gestational day (GD) 1 to GD 18.	• Pubertal onset delayed (vaginal opening, first estrus)↓; • Ovarian morphology & function impaired (weight↓, follicles↓, corpora lutea↓); • Reproductive hormone disruption (LH↓;impaired preovulatory surge);	PPARα activation (liver)→FGF21↑→Suppressed VAP neuronal activity (SCN)→Kisspeptin↓(AVPV)→GnRH/LH release↓→Delayed HPG axis activation & ovarian dysfunction.	(Zhang et al., 2021)

↑, increase; ↓, decrease; ↔, no significant change.

### 3.2 Metabolic reprogramming by PFAS: mediated by dual PPAR pathways

As evidenced in studies where PFOS (0.3 and 3 mg/g in male mice for 21 days) and PFOA (3.5 mg/kg)were administered, due to their structural similarity to fatty acids, PFAS can disrupt metabolic homeostasis in cells of the urinary system through the fatty acid mimicry effect (Lee et al., 2023; Zhang et al., 2023). A core mechanism of PFAS action involves the activation of peroxisome proliferator-activated receptors (PPARs). PFAS act as potent ligands for PPAR isoforms, particularly PPARα and PPARγ. Recent work by Boyd et al., investigating PFAS including PFOS, HQ-115, and GenX at concentrations ranging from 10 nM to 1 μM, demonstrated that PFAS exposure regulates PPAR target genes in testicular germ cell tumor cells, inducing reprogramming of steroidogenesis and lipid metabolism to promote tumorigenesis (Boyd et al., 2025). A systematic review indicates that PFAS, particularly PFOA and PFOS, activate PPARα, regulating downstream genes involved in lipid and glucose metabolism, inducing oxidative stress, and disrupting renal lipid homeostasis, which can lead to tubular injury. Long-term exposure is directly associated with an increased risk of renal cell carcinoma (Stanifer et al., 2018). Furthermore, PFAS, notably PFOS and PFOA, can synergize with factors such as a high-fat diet to dysregulate glucose, lipid, and amino acid metabolism in prostate cells through aberrant PPARα activation, thereby providing substrates for prostate cancer development. This synergy, particularly under PFOS exposure at 10 mg/kg/day, has been shown to accelerate prostate tumor growth *in vivo* (Imir et al., 2021; Hu et al., 2022). A study by Yang et al. demonstrated that PFOS activates PPARδ, leading to metabolic stress in mouse spermatocytes, which in turn sensitizes the inositol 1,4,5-trisphosphate receptor (IP3R), disrupts ER calcium homeostasis, depletes ER calcium stores, and triggers ER stress-mediated apoptosis (Yang et al., 2024). Research by Su et al. links PFOS exposure to renal impairment, suggesting that the disruption of PPAR signaling and lipid metabolism may initiate inflammatory responses that ultimately lead to renal damage (Su et al., 2025). Separately, Niu et al., in a study measuring specific PFAS (PFOA, PFOS, PFNA, PFHxS) at mean concentrations of 3.25, 13.81, 1.15, and 2.20 ng/dL respectively, found that PFAS significantly increases the risk of hyperuricemia, potentially through PPAR-related mechanisms affecting renal function, a hypothesis warranting further study (Niu et al., 2024).

In addition to PPAR-dependent pathways, PFAS exert metabolic disruption through PPAR-independent mechanisms. For example, PFOS and PFOA can directly and competitively bind to fatty acid transporters or key metabolic enzymes, thereby disrupting normal fatty acid β-oxidation and the tricarboxylic acid (TCA) cycle (Boyd et al., 2022; Boyd et al., 2024; Gabal et al., 2025). This leads to ROS accumulation, causing oxidative DNA damage and lipid peroxidation, and creating a persistent state of metabolic stress.

In summary, through these complementary PPAR-dependent and independent pathways, PFAS collectively disrupt the metabolic network and compromise the function of urinary system cells, creating a milieu conducive to carcinogenesis. Moreover, KEGG pathway enrichment analyses confirm that the toxicity of a broad set of 20 PFAS (e.g., PFOA, PFHxA, PFHxS, PFBA) involves key metabolic pathways, including PPAR signaling, insulin resistance, and AMPK signaling (Song et al., 2025). Molecular docking simulations further demonstrate a strong binding affinity of PFAS to core targets, including MMP-9, BCL-2, and CYP3A43, providing structural insights into their potential to disrupt metabolism.

### 3.3 Endocrine disruption: hormonal homeostasis dysregulation and functional impairment

As typical endocrine-disrupting chemicals (EDCs), PFAS, particularly PFOA and PFOS, disrupt systemic hormonal homeostasis and exert specific toxic effects on the structure and function of the urogenital system (Metcalfe et al., 2022). A core aspect of their disruptive mechanism involves anti-androgenic activity and indirect impact on hormone-sensitive organs, with the severity of outcomes being critically dependent on the timing of exposure. PFAS interfere with testosterone biosynthesis and exhibit significant anti-androgenic effects in the testes. Research indicates that these pollutants can inhibit the activity of key steroidogenic enzymes, including StAR and CYP17A1, in Leydig cells. This inhibition, observed following exposure to PFOA (up to 20 mg/kg/day), PFOS (up to 10 mg/kg/day), and PFHpA (up to 100 mg/kg/day), leads to reduced testosterone production (Kahn et al., 2020; Ajana et al., 2025). This disruption can impair the normal differentiation and development of the male reproductive system, with potential effects extending into adulthood. This is supported by a recent mouse study, in which 4-week oral exposure to PFO4DA (up to 10 mg/kg/day) was found to suppress testosterone synthesisby specifically downregulating StAR and CYP11A1 expression in Leydig cells, consequently impairing Sertoli cell function (Mutalifu et al., 2025). Given that Sertoli cells are crucial for maintaining the blood-testis barrier and the immune-privileged microenvironment of the seminiferous tubules, their dysfunction disrupts spermatogenesis, resulting in reduced sperm counts and increased sperm malformation (Law and Oatley, 2020; Heinrich et al., 2021; Washburn et al., 2022). Furthermore, PFAS can be detected directly in seminal plasma, with levels of PFOA showing a significant inverse correlation with sperm motility (Sánchez-Resino et al., 2023; Sun et al., 2023; Di Giorgi et al., 2024). The underlying mechanism may involve PFAS disrupting Sertoli cell tight junctions and directly interfering with sperm cell energy metabolism and membrane stability (Sun et al., 2023; Qu et al., 2025; Shi et al., 2025). Alterations in testosterone levels can also affect the prostate, a highly hormone-dependent organ, thereby increasing the risk of prostatic lesions (Wang et al., 2024; Feijó et al., 2025).

It is essential to note that the duration of exposure is a crucial factor in determining the severity of endocrine disruption caused by PFAS. The fetal period represents a critical window of organ development, during which even low-dose exposure may trigger irreversible changes. This is strongly supported by birth cohort studies linking prenatal PFAS exposure to altered anogenital distance (AGD) growth trajectories, with distinct sexual dimorphism. For instance, maternal exposure to even low levels of PFBS (geometric mean: 0.02 ng/mL) was associated with a 44% reduced odds of “rapid-growth” AGD in boys, while exposure to several other PFAS (e.g., PFHxS, PFTrDA) was associated with a ≥ 2-fold increase in the oddsof rapid-growth AGD in girls (Xi et al., 2025). Adolescence marks a second critical developmental window for the reproductive system. As demonstrated in C57BL/6 male mice exposed to a mixture of PFOS, PFOA, PFNA, PFHxS, and GenX (each at 20 µg/L), PFAS exposure disrupted the timing of pubertal onset and the differentiation of spermatogonial stem cells, resulting in long-term consequences for fertility (Maxwell et al., 2024). In contrast, exposure during adulthood predominantly induces reversible functional alterations rather than permanent structural damage (Niu et al., 2024).

In summary, PFAS disrupt urogenital hormonal balance and function primarily by interfering with hormone synthesis, metabolism, and signaling axes, with anti-androgenic effects constituting a dominant mechanism. The fetal and adolescent periods represent particularly vulnerable windows of exposure.

### 3.4 Epigenetic perturbations: dysregulation of gene expression and oncogenic memory

The key mechanism underlying the toxicity of PFAS to the urogenital system involves not direct mutagenic damage to DNA sequences, but rather the dysregulation of cellular gene expression programs via epigenetic modifications, which in turn drives the formation of precancerous lesions and carcinogenesis. This process is centered on the ability of PFAS to disrupt key epigenetic regulators, including DNA methylation patterns, histone modifications, and microRNA expression, collectively reshaping the epigenetic landscape of urogenital cells. Such perturbations can establish a stable, long-term “epigenetic memory” that may be propagated through cell divisions, thereby locking cells into a state of abnormal proliferation or heightened susceptibility to malignant transformation.

At the level of DNA methylation, exposure to PFAS can induce genome-wide methylation dysregulation. Studies have shown that acute PFOA exposure at doses of 1, 5, 10, or 20 mg/kg/dayleads to abnormal DNA methylation in mouse kidneys, with differentially methylated regions enriched in pathways associated with renal cancer (Rashid et al., 2020). PFAS promote tumorigenesis through mechanisms such as genome-wide hypomethylation and the hypermethylation of tumor suppressor genes like GSTP1. Additionally, studies in mice exposed to PFOS (at 5, 10, and 20 mg/kg/day) have shown that PFAS upregulates the expression of renal injury markers (e.g., Acta2, Bcl2l1) while reducing their DNA methylation, thereby facilitating renal fibrosis and carcinogenesis (Wen et al., 2022). In testicular tissue, PFOS disrupt the methylation status of imprinted genes (e.g., IGF2/H19), which can lead to abnormal differentiation of primordial germ cells. This is considered an essential mechanism for the increased risk of testicular germ cell tumors (TGCTs) (Kobayashi et al., 2017; Boyd et al., 2024). Research by Fazal et al. further indicates that PFAS exposure is associated with chemoresistance in TGCTs, and that such aberrant methylation patterns can be stably maintained in tumor cell lines (Fazal et al., 2021).

Beyond DNA methylation, histone modifications, and microRNA regulation are also crucial. The research team, led by Wen et al., discovered that PFOS can upregulate the expression of histone demethylases Kdm1a and Kdm4c in renal tissue, thereby inducing alterations in histone modifications. This epigenetic reprogramming indirectly affects transcription factors and pathways involved in renal injury, ultimately contributing to impaired renal function and an elevated tumorigenic risk (Wen et al., 2022). At population exposure levels (e.g., median concentrations of 0.10-3.70 ng/mL for prevalent PFAS like n-PFOA, n-PFOS, and PFHxS), PFAS dysregulates the expression of specific microRNAs. These non-coding RNAs function by inhibiting the translation of target mRNAs, and their dysregulation can suppress the expression of tumor suppressor genes, thereby promoting the abnormal proliferation of prostate and renal cells (Furlong et al., 2025). Recent studies have revealed more complex regulatory layers. For instance, both *in vitro* (25-50 µM) and *in vivo* (0.02 mg/kg/day) exposure models demonstrate that low-dose PFOA can bind to and inhibit the activity of the N6-methyladenosine (m6A) demethylase FTO. This increases the m6A modification level on MAPK15 mRNA, enhancing its stability and translation efficiency, which ultimately accelerates the proliferation, migration, and invasion of prostate cancer cells through the activation of the autophagic pathway (Qian et al., 2025). Furthermore, the direct disruptive effects of PFAS on cellular structures are closely linked to epigenetic alterations. Professor Gao S found that using 50 µM PFOS can disrupt the cytoskeleton in Sertoli cells by activating the JNK/p-JNK signaling pathway, thereby impairing blood-testis barrier function. This damage, potentially mediated through oxidative stress, may affect semen quality, suggesting a link to male infertility (Gao et al., 2024). Subsequent molecular docking studies revealed that PFAS, notably PFOA at serum concentrations exceeding 8 ng/mL, can directly perturb chromatin structure by interfering with DNA-protamine interactions to form stable ternary adducts, thereby physically compromising the stability of the epigenetic regulatory framework (Marinaro et al., 2025; Musella et al., 2025). This finding offers a novel mechanistic insight into understanding the reproductive toxicity of PFAS.

## 4 PFAS and MPs: potential for combined exposure and interaction

Although, as shown in **Table 3**, the toxic mechanisms and associated diseases of PFAS and MPs on the urogenital system are distinct, real-world exposure typically involves pollutant mixtures. Therefore, their combined health risks must be assessed on the basis of their co-occurrence in the environment (Barhoumi et al., 2022). Polytetrafluoroethylene (PTFE) is widely used as a water- and stain-resistant coating in everyday products such as non-stick cookware, waterproof clothing, and in specialized tapes. During use and abrasion, PTFE-based materials can degrade, releasing both fibrous MPs and leaching PFAS into the environment (Schellenberger et al., 2022; Kang et al., 2023). Wastewater treatment plants and landfills are major environmental sinks for both MPs and PFAS. These pollutants enter aquatic systems via domestic and industrial wastewater discharges. Of particular concern, if the resulting sewage sludge is applied to agricultural land as fertilizer, it can become a significant pathway for contaminating soil ecosystems (Ma et al., 2025). (**Figure 1**).

**Table 3:** Comparison of MPs and PFAS: characteristics and urotoxicity.

Comparison Parameters	Microplastics (MPs)	Per- and polyfluoroalkyl substances (PFAS)	Ref.
Basic Characteristics	Solid particulate pollutants. Chemically inert but often contain additives and adsorb environmental contaminants.	A class of stable, synthetic, fluorinated organic compounds.	(Dissanayake et al., 2022; Zheng et al., 2022; Dragon et al., 2023)
Main Exposure Pathways	• Ingestion:​ Contaminated food, water. • Inhalation:​ Airborne particles. • Medical Procedures/Implants: Exposure via intravenous infusions and dialysis supplies. • Maternal Transfer:Via breast milk.	• Ingestion:​ Contaminated food, water. • Inhalation:​ Airborne particles • Daily Use:​ Non-stick cookware, waterproof textiles. • Personal Care Products:Such as hygiene products, skin care products, make-up and perfumes. • Maternal Transfer:Via placenta and breast milk. • Medical Procedures/Implants: Exposure via catheters, stents, etc.	(Sunderland et al., 2019; Amobonye et al., 2021; Abraham and Monien, 2022; Ragusa et al., 2022; Zheng et al., 2022; Dewapriya et al., 2023; Passos et al., 2023; Céline et al., 2024; Briassoulis et al., 2025; Casella et al., 2025; Fillman et al., 2025; Yakovenko et al., 2025)
Key Mechanisms of Urotoxicity	•Oxidative Stress:Induces ROS burst, triggering downstream inflammatory responses. •Programmed Cell Death:Activates ferroptosis, directly leading to renal fibrosis and malignancy. •Cross-organ Communication:Disrupts the intestinal barrier, indirectly inducing renal toxicity. •Carrier & Synergistic Effects:Acts as a carrier, enhancing the toxicity of adsorbed pollutants.	•Metabolic Reprogramming:Dual PPAR pathways-mediated disruption of renal metabolic and redox homeostasis, leading to indirect renal injury. •Endocrine Disruption:Anti-androgenic effects that disturb hormonal homeostasis, impairing urogenital development and function. •Epigenetic Regulation:Induces epigenetic perturbations, causing gene expression dysregulation and increased long-term carcinogenic risk.	(Wang et al., 2021; Dissanayake et al., 2022; Goodman et al., 2022; Wang et al., 2022; Zou et al., 2022; Cheng et al., 2024; He et al., 2024; Liang et al., 2024; Li et al., 2024) vs (Kahn et al., 2020; Law and Oatley, 2020; Wen et al., 2022; Lee et al., 2023; Zhang et al., 2023; Ajana et al., 2025)
Associated Diseases	•Kidney injury; Fibrosis. •Cancers: Prostate, Penile, Renal. •Male Infertility: Testicular injury, impaired spermatogenesis. •Hypertension post-kidney transplant.	•Kidney stones; Hyperuricemia. •Kidney injury; Fibrosis. •Cancers: Prostate, Renal, Testicular germ cell tumors. •Male Infertility: Testicular injury, impaired spermatogenesis.	(Wang et al., 2024; Li et al., 2025; Wang et al., 2025; Wang et al., 2025; Zhao et al., 2025; Zhou et al., 2025) vs (Rashid et al., 2020; Imir et al., 2021; Wen et al., 2022; Sun et al., 2023; Boyd et al., 2024; Niu et al., 2024; Chen et al., 2025; Su et al., 2025)

The mechanisms summarized above are supported by extensive studies, including but not limited to (Wang et al., 2021; Dissanayake et al., 2022; Goodman et al., 2022; Wang et al., 2022; Zou et al., 2022; Cheng et al., 2024; He et al., 2024; Liang et al., 2024; Li et al., 2024) for MPs and (Kahn et al., 2020; Law and Oatley, 2020; Wen et al., 2022; Lee et al., 2023; Zhang et al., 2023; Ajana et al., 2025) for PFAS. For a detailed discussion, see Sections 2 and 3 in the main text.

Extensive research has confirmed interfacial interactions between PFAS and MPs, primarily characterized by the adsorption of PFAS onto MP surfaces and their consequent co-transport. The initial migration and retention of PFAS molecules at the liquid-solid interface of MPs is termed “adsorption”, while diffusion into the polymer matrix is “absorption” (Vieira et al., 2021). This adsorption process is governed by a combination of electrostatic forces, hydrophobic interactions, the physicochemical properties of the pollutants, and environmental conditions (Ateia et al., 2020; Min et al., 2020; Bhagwat et al., 2021; Meng et al., 2023; Kang et al., 2024; Soltanighias et al., 2024). However, the “carrier effect” is not equivalent to “biological synergistic effect.” According to the principles of mixture toxicology, their co-exposure may result in different biological interaction modes—such as synergistic, additive, or antagonistic effects—the specific manifestation of which highly depends on the exposure conditions and biological system. This carrier effect can lead to an increased composite pollution burden (Jian et al., 2024; Yu et al., 2024; Sands et al., 2025).

### 4.1 Interaction mechanisms and potential effect patterns

Limited existing studies have suggested several possible interaction pathways and corresponding effects. First, synergistic effects represent the biological effect mode with the highest occurrence potential, yet their manifestation is highly condition-dependent. The underlying mechanisms may encompass two main aspects: one involvestoxicokinetic modulation.A mouse study by Zhou P et al. investigated co-exposure to MPs (PS and PBS) and PFAS (legacy: PFOA, PFOS; alternatives: HFPO-TA, 6:2 FTSA) (Zhou et al., 2025). MPs, particularly PS, increased serum albumin and downregulated hepatic efflux transporters (Mrp2, Mrp4), inhibiting PFAS elimination. This led to significantly increased PFAS bioavailability and reduced fecal excretion. Molecular docking indicated legacy PFAS had higher binding affinity to these proteins, rendering them more susceptible to MP-mediated modulation than alternatives. While the other involvesadditive and amplified biological stress. Mia Sands and colleagues, has directly addressed their combined effects on this system (Sands et al., 2025). That pioneering work confirmed that co-exposure to PFAS and MPs activates pathways associated with oxidative stress, antioxidant response, and DNA damage repair across several human-derived cell lines. Notably, the toxic effects displayed apparent cell-type specificity. A significant synergistic toxicity was observed in the renal cell line A498, whereas the synergistic effect was considerably weaker in the prostate cancer cell line PC3. As the first study in this field, it provided initial evidence suggesting the potential for synergistic toxicity from PFAS-MP mixtures in human cellular models. Second, antagonistic effects are also possible. Liu et al. reported that PS-MPs interacted with PFOS to produce antagonism in plants, likely due to PFOS adsorption onto MPs reducing its bioavailability and acute toxicity (e.g., oxidative stress) (Liu et al., 2023). Aquatic studies confirm MPs can strongly adsorb PFAS, reducing aqueous “free” concentrations and creating exposure-level antagonism (Jian et al., 2024). However, this reduced free fraction does not always translate to lower overall toxicity, and some endpoints may be exacerbated, suggesting antagonism may be limited to the exposure medium. Finally, in the absence of strong interactions, MPs and PFAS may produce approximately additive effects via shared mechanisms like oxidative stress and inflammation, disrupting tissue homeostasis. Existing reviews note the condition-dependent diversity of co-exposure outcomes, implying additivity should be treated as a baseline hypothesis requiring empirical validation (Yu et al., 2024).

### 4.2 Weight of evidence, limitations, and key uncertainties

Current research on the combined toxicity of MPs and PFAS has predominantly focused on aquatic organisms (Rainieri et al., 2018; Yang et al., 2020; Álvarez-Ruiz et al., 2021; Islam et al., 2021; Chen et al., 2022; Soltanighias et al., 2024; Yu et al., 2024). By contrast, studies investigating the impacts of co-exposure on the human urogenital system are incredibly scarce. The few existing studies have mostly been *in vitro* cell experiments or animal models investigating the effects of co−exposure to the two pollutants on urogenital system endpoints (Liu et al., 2025; Sands et al., 2025; Zhou et al., 2025). In human studies, cohort data that simultaneously measure both MPs and PFAS exposure and link them to health outcomes are extremely scarce, making it difficult to assess health risks under real−world co−exposure scenarios.

This research faces multiple challenges. First, the detection of MPs in human samples (e.g., urine, semen) is highly vulnerable to experimental contamination, and differences in particle size definition and quantification methods across analytical platforms result in low comparability between studies. Second, animal studies often use relatively high doses of MPs to elucidate mechanisms, which deviates from actual human exposure levels; this gap requires bridging via toxicokinetic modeling and long−term, low−dose studies. Furthermore, most existing research has focused on legacy PFAS (e.g., PFOA/PFOS), whereas real−world mixtures include a complex variety of PFAS alternatives and precursors whose interactions with MPs may differ and remain to be systematically evaluated.

In summary, the interactive effects (synergistic, additive, or antagonistic) are highly dependent on multiple factors, including particle size and polymer type of MPs, chain length and class of PFAS, exposure dose and ratio, exposure route, and host status. Therefore, there is insufficient evidence to conclude that combined toxicity is necessarily stronger than single exposure. The main significance of current studies is to highlight the potential for interactions and complex mechanisms, rather than to provide definitive conclusions.

## 5 Discussion

This review systematically examines the potential threats posed by environmental pollutants, specifically PFAS and MPs, to urogenital health and their underlying mechanisms. By analyzing the urotoxicity of MPs and PFAS separately, it is evident that both pollutant classes can damage key urogenital organs, such as the kidneys, through distinct pathways. MP-induced toxicity is initiated by oxidative stress as a central trigger, which subsequently activates downstream effector pathways, including programmed cell death and the NLRP3 inflammasome, leading to renal tubular epithelial injury. Furthermore, MPs can exert cross-organ effects by disrupting the gut-kidney axis. Their toxicity exhibits size-dependent characteristics, and MPs can act as carriers, synergizing with other pollutants via a “Trojan horse” effect. PFAS preferentially accumulate in the kidneys, inducing long-term metabolic reprogramming via both PPAR-dependent and PPAR-independent pathways, thereby disrupting lipid and energy homeostasis. As typical endocrine-disrupting chemicals, PFAS indirectly dysregulate hormonal balance, primarily through core mechanisms such as anti-androgenic effects, with the severity of urogenital damage being contingent upon the exposure window. Additionally, PFAS can cause epigenetic perturbations, leading to aberrant gene expression and conferring long-term carcinogenic risk.

Compared with studies on single exposure, investigations into the combined effects of PFAS and MPs are of greater real−world relevance. Exposure in the actual environment frequently occurs as mixtures of pollutants. PFAS and MPs commonly coexist in environmental matrices, and MPs can act as carriers for PFAS via interfacial interactions, forming “pollutant complexes” that undergo co−transport. However, the physical “carrier effect” does not equate to a fixed enhancement of biological toxicity. According to the principles of mixture toxicology, combined exposure may trigger distinct biological responses, including synergistic, additive, or antagonistic effects. The specific outcome is highly dependent on multiple factors, such as the physicochemical properties of MPs, the type and structure of PFAS, exposure dose and ratio, and the biological system itself. Limited current studies have revealed the complex and condition−dependent nature of such interactions. Combined exposure may lead to divergent outcomes through multiple mechanisms: for instance, MPs may induce synergistic toxicity by modulating the bioavailability of PFAS or by amplifying cellular stress responses; alternatively, adsorption of PFAS onto MPs may reduce their free concentration, resulting in antagonistic effects. Furthermore, when the interaction is not significant, the two pollutants may also produce approximately additive effects through common pathways such as oxidative stress and inflammation. It must be clearly recognized that the evidence base in this field remains weak. Most existing studies focus on aquatic models or high−dose animal experiments, which deviate from the real scenario of long−term exposure in human populations. Epidemiological evidence that simultaneously characterizes co−exposure and links it to health outcomes at the population level is particularly scarce. Therefore, the main significance of current work lies in revealing the potential for interactions between the two pollutants and the diversity of mechanisms that may lead to complex toxicological effects, rather than confirming that their combined toxicity is necessarily stronger.

Future research should strive to establish a complete evidence chain spanning from environmental co−occurrence to health risk assessment. First, develop standardized methods for internal exposure characterization. Analytical techniques should be established for the simultaneous and accurate quantification of MPs (especially nanoscale plastics) and multiple PFAS in human tissue samples. In addition, MP surrogates that can simulate real−world environmental aging processes should be developed to accurately characterize the morphology and bioaccessibility of “pollutant complexes”. Second, elucidate the toxicokinetic mechanisms of interactions and construct predictive models. Using microphysiological systems (such as kidney organoids and blood−testis barrier models) and animal models, systematically resolve how MPs acting as carriers modulate the absorption, distribution, and excretion of PFAS. Multi−omics technologies should be applied to reveal the molecular interaction networks underlying synergistic or antagonistic effects on shared pathways (e.g., oxidative stress). Integrating these data, physiologically based pharmacokinetic (PBPK) and adverse outcome pathway (AOP) models should be constructed to predict risks under different exposure scenarios. Finally, generate key population−based epidemiological evidence and promote translational risk management. Prospective cohort studies should be launched to longitudinally track the associations between co−exposure levels of MPs and PFAS and urogenital health outcomes within the same population. Such evidence forms the foundation for eventually developing a risk assessment framework for mixed pollutant exposure and providing direct scientific support for the regulation of related products.

## 6 Conclusion

Although the individual genitourinary toxicity mechanisms of PFAS and MPs have been partially elucidated, research on the health impacts of their combined exposure remains in the preliminary stage, with substantial knowledge gaps. Existing evidence suggests that combined exposure may trigger distinct outcomes including synergistic, additive, or antagonistic effects through complex mechanisms such as the carrier effect, toxicokinetic modulation, and activation of common pathways. The specific patterns and consequences are highly dependent on the physicochemical properties of the pollutants, exposure scenarios, and the biological system. To overcome current limitations in understanding, future research must integrate multidisciplinary approaches spanning environmental science, analytical chemistry, toxicology, and epidemiology, with coordinated efforts in three core areas: precise characterization of internal exposure, multi-scale mechanistic dissection, and population-based empirical studies. This represents not only the essential path to deepen mechanistic insights but also the key to transforming the risk assessment paradigm from single-pollutant evaluation to combined-exposure assessment, ultimately providing a robust scientific basis for the development of targeted risk management and control strategies.

## References

[B1] Abraham K. Monien B. H. (2022). Transdermal absorption of (13)C(4)-perfluorooctanoic acid ((13)C(4)-PFOA) from a sunscreen in a male volunteer - What could be the contribution of cosmetics to the internal exposure of perfluoroalkyl substances (PFAS)? Environ. Int. 169, 107549. doi: 10.1016/j.envint.2022.107549. PMID: 36191486

[B2] Ahmed Y. H. El-Naggar M. E. Rashad M. M. A M. Y. Galal M. K. Bashir D. W. (2022). Screening for polystyrene nanoparticle toxicity on kidneys of adult male albino rats using histopathological, biochemical, and molecular examination results. Cell Tissue Res. 388, 149–165. doi: 10.1007/s00441-022-03581-5. PMID: 35088181 PMC8976822

[B3] Ajana R. Rachoń D. Gałęzowska G. (2025). Reproductive toxicity of per- and polyfluoroalkyl substances. Environ. Toxicol. Pharmacol. 117, 104740. doi: 10.1016/j.etap.2025.104740. PMID: 40473150

[B4] Álvarez-Ruiz R. Picó Y. Campo J. (2021). Bioaccumulation of emerging contaminants in mussel (Mytilus galloprovincialis): influence of microplastics. Sci. Total Environ. 796, 149006. doi: 10.1016/j.scitotenv.2021.149006. PMID: 34328891

[B5] Amobonye A. Bhagwat P. Raveendran S. Singh S. Pillai S. (2021). Environmental impacts of microplastics and nanoplastics: a current overview. Front. Microbiol. 12. doi: 10.3389/fmicb.2021.768297. PMID: 34975796 PMC8714882

[B6] Ateia M. Zheng T. Calace S. Tharayil N. Pilla S. Karanfil T. (2020). Sorption behavior of real microplastics (MPs): insights for organic micropollutants adsorption on a large set of well-characterized MPs. Sci. Total Environ. 720, 137634. doi: 10.1016/j.scitotenv.2020.137634. PMID: 32146408

[B7] Barhoumi B. Sander S. G. Tolosa I. (2022). A review on per- and polyfluorinated alkyl substances (PFASs) in microplastic and food-contact materials. Environ. Res. 206, 112595. doi: 10.1016/j.envres.2021.112595. PMID: 34929191

[B8] Bhagwat G. Tran T. K. A. Lamb D. Senathirajah K. Grainge I. O’Connor W. . (2021). Biofilms enhance the adsorption of toxic contaminants on plastic microfibers under environmentally relevant conditions. Environ. Sci. Technol. 55, 8877–8887. doi: 10.1021/acs.est.1c02012. PMID: 34152751

[B9] Boyd R. I. Ahmad S. Singh R. Fazal Z. Prins G. S. Madak Erdogan Z. . (2022). Toward a mechanistic understanding of poly- and perfluoroalkylated substances and cancer. Cancers (Basel) 14, 2919. doi: 10.3390/cancers14122919. PMID: 35740585 PMC9220899

[B10] Boyd R. I. Shokry D. Fazal Z. Rennels B. C. Freemantle S. J. La Frano M. R. . (2024). Perfluorooctanesulfonic acid alters pro-cancer phenotypes and metabolic and transcriptional signatures in testicular germ cell tumors. Toxics 12, 232. doi: 10.3390/toxics12040232. PMID: 38668455 PMC11054796

[B11] Boyd R. I. Shokry D. Rennels B. C. Adam Y. Powell C. Johnson S. . (2025). The role of lipid metabolism and peroxisome proliferator activation in mediating pro-cancer phenotypes of poly- and perfluoroalkyl substances in testicular cancer. Environ. Toxicol. Pharmacol. 120, 104866. doi: 10.1016/j.etap.2025.104866. PMID: 41237835 PMC13371115

[B12] Briassoulis G. Ilia S. Briassouli E. (2025). Exposure to per- and polyfluoroalkyl substances (PFASs) in healthcare: Environmental and clinical insights. Life. (Basel) 15, 1057. doi: 10.3390/life15071057. PMID: 40724559 PMC12298592

[B13] Butzlaff A. H. Deighton J. Le T. Brougham A. Bessler S. M. McKnight T. . (2025). PFAS, 6-PPD-Q, and microplastics in urban sewer overflows: co-occurrence and high-rate treatment assessment. NPJ Emerging Contaminants 1, 5. doi: 10.1038/s44454-025-00010-4. PMID: 41862587

[B14] Casella C. Cornelli U. Zanoni G. Moncayo P. Ramos-Guerrero L. (2025). Health risks from microplastics in intravenous infusions: evidence from Italy, Spain, and Ecuador. Toxics 13, 597. doi: 10.3390/toxics13070597. PMID: 40711041 PMC12298248

[B15] Céline C. Catherine B. Romane C. Laurence C. (2024). Per- and polyfluoroalkyls used as cosmetic ingredients - Qualitative study of 765 cosmetic products. Food Chem. Toxicol. 187, 114625. doi: 10.1016/j.fct.2024.114625, PMID: 38582342

[B16] Chen L. Han B. Yang S. Guo L. Zhao L. Liu P. . (2025). Toxicological effects and mechanisms of renal injury induced by inhalation exposure to airborne nanoplastics. J. Hazard. Mater. 488, 137393. doi: 10.1016/j.jhazmat.2025.137393. PMID: 39892132

[B17] Chen C. C. Shi Y. Zhu Y. Zeng J. Qian W. Zhou S. . (2022). Combined toxicity of polystyrene microplastics and ammonium perfluorooctanoate to Daphnia magna: mediation of intestinal blockage. Water Res. 219, 118536. doi: 10.1016/j.watres.2022.118536. PMID: 35550964

[B18] Chen J. Zhang Z. Xu G. Tang Q. Nie Z. Qiu J. . (2025). Exploring the impact of PFAS exposure and sleep duration on kidney stone formation in the U.S. population. Front. Public Health 13. doi: 10.3389/fpubh.2025.1606191. PMID: 40535425 PMC12174110

[B19] Cheng Y. Mai L. Lu X. Li Z. Guo Y. Chen D. . (2021). Occurrence and abundance of poly- and perfluoroalkyl substances (PFASs) on microplastics (MPs) in Pearl River Estuary (PRE) region: spatial and temporal variations. Environ. pollut. 281, 117025. doi: 10.1016/j.envpol.2021.117025. PMID: 33813193

[B20] Cheng W. Zhou Y. Chen H. Wu Q. Li Y. Wang H. . (2024). The iron matters: Aged microplastics disrupted the iron homeostasis in the liver organoids. Sci. Total Environ. 906, 167529. doi: 10.1016/j.scitotenv.2023.167529. PMID: 37788777

[B21] Dewapriya P. Chadwick L. Gorji S. G. Schulze B. Valsecchi S. Samanipour S. . (2023). Per- and polyfluoroalkyl substances (PFAS) in consumer products: Current knowledge and research gaps. J. Hazard. Mater. Lett. 4, 100086. doi: 10.2139/ssrn.4449433. PMID: 40330906

[B22] Di Giorgi A. Basile G. Bertola F. Tavoletta F. Busardò F. P. Tini A. (2024). A green analytical method for the simultaneous determination of 17 perfluoroalkyl substances (PFAS) in human serum and semen by ultra-performance liquid chromatography-tandem mass spectrometry (UPLC-MS/MS). J. Pharm. Biomed. Anal. 246, 116203. doi: 10.1016/j.jpba.2024.116203. PMID: 38759320

[B23] Dissanayake P. D. Kim S. Sarkar B. Oleszczuk P. Sang M. K. Haque M. N. . (2022). Effects of microplastics on the terrestrial environment: A critical review. Environ. Res. 209, 112734. doi: 10.1016/j.envres.2022.112734. PMID: 35065936

[B24] Dou J. Y. Liu S. Wang C. Y. Dai X. Lian L. H. Cui Z. Y. . (2026). Microplastics and nanoplastics, emerging pollutants, increased the risk of pulmonary fibrosis *in vivo* and *in vitro*: a comparative evaluation of their potential toxicity effects with different polymers and size. Toxicology 519, 154304. doi: 10.1016/j.tox.2025.154304. PMID: 41075967

[B25] Dragon J. Hoaglund M. Badireddy A. R. Nielsen G. Schlezinger J. Shukla A. (2023). Perfluoroalkyl substances (PFAS) affect inflammation in lung cells and tissues. Int. J. Mol. Sci. 24, 8539. doi: 10.3390/ijms24108539. PMID: 37239886 PMC10218140

[B26] Fazal Z. Singh R. Fang F. Bikorimana E. Baldwin H. Corbet A. . (2021). Hypermethylation and global remodelling of DNA methylation is associated with acquired cisplatin resistance in testicular germ cell tumours. Epigenetics 16, 1071–1084. doi: 10.1080/15592294.2020.1834926. PMID: 33126827 PMC8510620

[B27] Feijó M. Carvalho T. M. A. Fonseca L. R. S. Vaz C. V. Pereira B. J. Cavaco J. E. B. . (2025). Endocrine-disrupting chemicals as prostate carcinogens. Nat. Rev. Urol. 22, 609–631. doi: 10.1038/s41585-025-01031-9, PMID: 40379948

[B28] Feng L. Chen C. Xiong X. Wang X. Li X. Kuang Q. . (2024). PS-MPs promotes the progression of inflammation and fibrosis in diabetic nephropathy through NLRP3/Caspase-1 and TGF-β1/Smad2/3 signaling pathways. Ecotoxicol. Environ. Saf. 273, 116102. doi: 10.1016/j.ecoenv.2024.116102. PMID: 38382346

[B29] Fillman T. Coffin S. Ta B. Beglarian E. Linck W. Baek H. G. . (2025). Associations between PFAS in public water system drinking water and serum among Southern California adults. J. Expo Sci. Environ. Epidemiol. 36, 311–321. doi: 10.1038/s41370-025-00817-8. PMID: 41168418 PMC12960233

[B30] Furlong M. A. Liu T. Jung A. Beitel S. Hughes J. Krause R. . (2025). Per- and polyfluoroalkyl substances (PFAS) and microRNA: an epigenome-wide association study in firefighters. Environ. Res. 279, 121766. doi: 10.1016/j.envres.2025.121766. PMID: 40350013 PMC12203895

[B31] Gabal E. Azaizeh M. Baloni P. (2025). Investigating lipid and energy dyshomeostasis induced by per- and polyfluoroalkyl substances (PFAS) congeners in mouse model using systems biology approaches. Metabolites 15, 499. doi: 10.3390/metabo15080499. PMID: 40863118 PMC12388551

[B32] Gao S. Chen Z. Wu X. Wang L. Bu T. Li L. . (2024). Perfluorooctane sulfonate-induced Sertoli cell injury through c-Jun N-terminal kinase: a study by RNA-Seq. Am. J. Physiol. Cell Physiol. 327, C291–c309. doi: 10.1152/ajpcell.00212.2024. PMID: 38826136

[B33] Gao D. Zhang C. Guo H. Xu H. Liu H. Wang Z. . (2024). Low-dose polystyrene microplastics exposure impairs fertility in male mice with high-fat diet-induced obesity by affecting prostate function. Environ. pollut. 346, 123567. doi: 10.2139/ssrn.4548331. PMID: 38367694

[B34] Ghosh S. S. Wang J. Yannie P. J. Ghosh S. (2020). Intestinal barrier dysfunction, LPS translocation, and disease development. J. Endocr. Soc 4, bvz039. doi: 10.1210/jendso/bvz039. PMID: 32099951 PMC7033038

[B35] Golden-Mason L. Salomon M. P. Matsuba C. Wang Y. Setiawan V. W. Chatzi L. . (2025). Assessing the impact of perfluoroalkyl substances on liver health: a comprehensive study using multi-donor human liver spheroids. Environ. Int. 203, 109763. doi: 10.1016/j.envint.2025.109763. PMID: 40914107 PMC12459911

[B36] Gong X. Zhou H. Huang Q. (2025). Assessing AlphaFold 3 for per- and polyfluoroalkyl substances docking in protein structures. Environ. Sci. Technol. 59, 18440–18449. doi: 10.1021/acs.est.5c03917. PMID: 40857166 PMC12424161

[B37] Goodman K. E. Hua T. Sang Q. A. (2022). Effects of polystyrene microplastics on human kidney and liver cell morphology, cellular proliferation, and metabolism. ACS Omega 7, 34136–34153. doi: 10.1021/acsomega.2c03453. PMID: 36188270 PMC9520709

[B38] Guo Y. Rong M. Fan Y. Teng X. Jin L. Zhao Y. (2025). The presence of microplastics in human semen and their associations with semen quality. Toxics 13, 566. doi: 10.3390/toxics13070566. PMID: 40711011 PMC12299061

[B39] Guo C. Zhang S. Zhang H. Chen L. Gao S. Xu M. . (2025). Per- and polyfluoroalkyl substances (PFAS) disrupt gut microbiome composition and metabolism in metabolic syndrome: evidence from a host-free *in vitro* colonic model. Environ. pollut. 386, 127189. doi: 10.1016/j.envpol.2025.127189. PMID: 41038555

[B40] He S. Cai J. Jia T. Mao Z. Zhou L. Zhang X. . (2024). New sight of renal toxicity caused by UV-aged polystyrene nanoplastics: Induced ferroptosis via adsorption of transferrin. Small 20, e2309369. doi: 10.1002/smll.202309369. PMID: 38175859

[B41] He L. Liu C. Zhao H. Yang Z. Zhou E. Chen H. . (2025). Unveiling the hidden threat: per- and polyfluoroalkyl substance pollution’s distinct traits and crucial impacts on lung function in Chinese plateau populations. Environ. Health (Wash) 3, 605–615. doi: 10.1021/envhealth.4c00268. PMID: 40567271 PMC12186208

[B42] Heinrich A. Bhandary B. Potter S. J. Ratner N. DeFalco T. (2021). Cdc42 activity in Sertoli cells is essential for maintenance of spermatogenesis. Cell Rep. 37, 109885. doi: 10.1016/j.celrep.2021.109885. PMID: 34706238 PMC8604081

[B43] Hong R. Shi Y. Fan Z. Gao Y. Chen H. Pan C. (2024). Chronic exposure to polystyrene microplastics induces renal fibrosis via ferroptosis. Toxicology 509, 153996. doi: 10.1016/j.tox.2024.153996. PMID: 39532264

[B44] Horvatits T. Tamminga M. Liu B. Sebode M. Carambia A. Fischer L. . (2022). Microplastics detected in cirrhotic liver tissue. EBioMedicine 82, 104147. doi: 10.1016/j.ebiom.2022.104147. PMID: 35835713 PMC9386716

[B45] Hu W. Y. Lu R. Hu D. P. Imir O. B. Zuo Q. Moline D. . (2022). Per- and polyfluoroalkyl substances target and alter human prostate stem-progenitor cells. Biochem. Pharmacol. 197, 114902. doi: 10.1016/j.bcp.2021.114902. PMID: 34968493 PMC8890783

[B46] Huang H. M. Peng H. L. Huang C. M. Zhang J. T. Li Y. H. Lin Z. L. . (2025). Melatonin alleviates the damage of polystyrene microplastics to porcine oocytes by reducing oxidative stress and mitochondrial damage, and regulating autophagy and apoptosis levels. Anim. (Basel) 15, 3163. doi: 10.3390/ani15213163. PMID: 41227494 PMC12607468

[B47] Imir O. B. Kaminsky A. Z. Zuo Q. Y. Liu Y. J. Singh R. Spinella M. J. . (2021). Per- and polyfluoroalkyl substance exposure combined with high-fat diet supports prostate cancer progression. Nutrients 13, 3902. doi: 10.3390/nu13113902. PMID: 34836157 PMC8623692

[B48] Islam N. Garcia da Fonseca T. Vilke J. Gonçalves J. M. Pedro P. Keiter S. . (2021). Perfluorooctane sulfonic acid (PFOS) adsorbed to polyethylene microplastics: accumulation and ecotoxicological effects in the clam Scrobicularia plana. Mar. Environ. Res. 164, 105249. doi: 10.1016/j.marenvres.2020.105249. PMID: 33477023

[B49] Jeon B. J. Ko Y. J. Cha J. J. Kim C. Seo M. Y. Lee S. H. . (2024). Examining the relationship between polystyrene microplastics and male fertility: Insights from an *in vivo* study and *in vitro* Sertoli cell culture. J. Korean Med. Sci. 39, e259. doi: 10.3346/jkms.2024.39.e259. PMID: 39376189 PMC11458380

[B50] Jian M. Chen X. Liu S. Liu Y. Liu Y. Wang Q. . (2024). Combined exposure with microplastics increases the toxic effects of PFOS and its alternative F-53B in adult zebrafish. Sci. Total Environ. 920, 170948. doi: 10.1016/j.scitotenv.2024.170948. PMID: 38365036

[B51] Jiang N. Zheng X. Zhang N. Cao Y. (2025). The detrimental effects of microplastic exposure on kidney function. Front. Med. (Lausanne) 12. doi: 10.3389/fmed.2025.1620733. PMID: 41064517 PMC12500543

[B52] Kahn L. G. Philippat C. Nakayama S. F. Slama R. Trasande L. (2020). Endocrine-disrupting chemicals: implications for human health. Lancet Diabetes Endocrinol. 8, 703–718. doi: 10.1016/s2213-8587(20)30129-7. PMID: 32707118 PMC7437820

[B53] Kang P. Zhao Y. Wei T. Cai Y. Ji B. Addo-Bankas O. (2024). Interactions between MPs and PFASs in aquatic environments: a dual-character situation. J. Environ. Manage. 351, 119907. doi: 10.1016/j.jenvman.2023.119907. PMID: 38157575

[B54] Kang P. Zhao Y. Zuo C. Cai Y. Shen C. Ji B. . (2023). The unheeded inherent connections and overlap between microplastics and poly- and perfluoroalkyl substances: a comprehensive review. Sci. Total Environ. 878, 163028. doi: 10.1016/j.scitotenv.2023.163028. PMID: 36963676

[B55] Kim Y. R. White N. Bräunig J. Vijayasarathy S. Mueller J. F. Knox C. L. . (2020). Per- and poly-fluoroalkyl substances (PFASs) in follicular fluid from women experiencing infertility in Australia. Environ. Res. 190, 109963. doi: 10.1016/j.envres.2020.109963. PMID: 32745751

[B56] Kobayashi S. Azumi K. Goudarzi H. Araki A. Miyashita C. Kobayashi S. . (2017). Effects of prenatal perfluoroalkyl acid exposure on cord blood IGF2/H19 methylation and ponderal index: the Hokkaido Study. J. Expo Sci. Environ. Epidemiol. 27, 251–259. doi: 10.1038/jes.2016.50. PMID: 27553991

[B57] Kuang Q. Gao L. Feng L. Xiong X. Yang J. Zhang W. . (2024). Toxicological effects of microplastics in renal ischemia-reperfusion injury. Environ. Toxicol. 39, 2350–2362. doi: 10.1002/tox.24115. PMID: 38156432

[B58] Landrigan P. J. Dunlop S. Treskova M. Raps H. Symeonides C. Muncke J. . (2025). The lancet countdown on health and plastics. Lancet 406, 1044–1062. doi: 10.1016/s0140-6736(25)01447-3. PMID: 40769171

[B59] La Porta E. Exacoustos O. Lugani F. Angeletti A. Chiarenza D. S. Bigatti C. . (2023). Microplastics and kidneys: an update on the evidence for deposition of plastic microparticles in human organs, tissues and fluids and renal toxicity concern. Int. J. Mol. Sci. 24, 14391. doi: 10.3390/ijms241814391. PMID: 37762695 PMC10531672

[B60] Law N. C. Oatley J. M. (2020). Developmental underpinnings of spermatogonial stem cell establishment. Andrology 8, 852–861. doi: 10.1111/andr.12810. PMID: 32356598 PMC8324036

[B61] Lee W. K. Lam T. K. Y. Tang H. C. Ho T. C. Wan H. T. Wong C. K. C. (2023). PFOS-elicited metabolic perturbation in liver and fatty acid metabolites in testis of adult mice. Front. Endocrinol. (Lausanne) 14. doi: 10.3389/fendo.2023.1302965. PMID: 38075064 PMC10703039

[B62] Li J. Deng C. Zou W. Jiang X. Dong Y. Yan Z. . (2025). Low-dose polystyrene microplastics exposure promotes human prostate cancer cell proliferation via GPX4-mediated ferroptosis. Ecotoxicol. Environ. Saf. 306, 119285. doi: 10.1016/j.ecoenv.2025.119285. PMID: 41151287

[B63] Li S. Gu X. Zhang M. Jiang Q. Xu T. (2024). Di (2-ethylhexyl) phthalate and polystyrene microplastics co-exposure caused oxidative stress to activate NF-κB/NLRP3 pathway aggravated pyroptosis and inflammation in mouse kidney. Sci. Total Environ. 926, 171817. doi: 10.1016/j.scitotenv.2024.171817. PMID: 38513858

[B64] Li Y. Li Y. Li J. Song Z. Zhang C. Guan B. (2023). Toxicity of polystyrene nanoplastics to human embryonic kidney cells and human normal liver cells: effect of particle size and Pb(2+) enrichment. Chemosphere 328, 138545. doi: 10.1016/j.chemosphere.2023.138545. PMID: 37011817

[B65] Li Z. Xu T. Peng L. Tang X. Chi Q. Li M. . (2023). Polystyrene nanoplastics aggravates lipopolysaccharide-induced apoptosis in mouse kidney cells by regulating IRE1/XBP1 endoplasmic reticulum stress pathway via oxidative stress. J. Cell. Physiol. 238, 151–164. doi: 10.1002/jcp.30913. PMID: 36370432

[B66] Liang Y. Liu D. Zhan J. Liu X. Li P. Ma X. . (2024). Polystyrene microplastics induce kidney injury via gut barrier dysfunction and C5a/C5aR pathway activation. Environ. pollut. 342, 122909. doi: 10.1016/j.envpol.2023.122909. PMID: 38036092

[B67] Lin Z. Li Z. Ji S. Lo H. S. Billah B. Sharmin A. . (2024). Size-dependent deleterious effects of nano- and microplastics on sperm motility. Toxicology 506, 153834. doi: 10.1016/j.tox.2024.153834. PMID: 38763425

[B68] Liu Y. Jin T. Wang L. Tang J. (2023). Polystyrene micro and nanoplastics attenuated the bioavailability and toxic effects of perfluorooctane sulfonate (PFOS) on soybean (Glycine max) sprouts. J. Hazard. Mater. 448, 130911. doi: 10.1016/j.jhazmat.2023.130911. PMID: 36860033

[B69] Liu Q. Ma X. Liu J. Liu Y. (2025). Multi-organ toxicity of combined PFOS/PS exposure and its application in network toxicology. Biol. (Basel) 14, 1714. doi: 10.3390/biology14121714. PMID: 41463487 PMC12730987

[B70] Liu D. Yan S. Wang P. Chen Q. Liu Y. Cui J. . (2023). Perfluorooctanoic acid (PFOA) exposure in relation to the kidneys: a review of current available literature. Front. Physiol. 14. doi: 10.3389/fphys.2023.1103141. PMID: 36776978 PMC9909492

[B71] Lv J. Liu G. Wang Z. Zhang J. Li Y. Wang Y. . (2025). Internalized polystyrene nanoplastics trigger testicular damage and promote ferroptosis via CISD1 downregulation in mouse spermatocyte. J. Nanobiotechnology 23, 537. doi: 10.1186/s12951-025-03620-7. PMID: 40707955 PMC12288249

[B72] Ma M. Coulon F. Tang Z. Hu Z. Bi Y. Huo M. . (2025). Unveiling the truth of interactions between microplastics and per- and polyfluoroalkyl substances (PFASs) in wastewater treatment plants: microplastics as a carrier of PFASs and beyond. Environ. Sci. Technol. 59, 2211–2221. doi: 10.1021/acs.est.4c08898. PMID: 39760641

[B73] Marinaro C. Bianchi A. R. Guerretti V. Barricelli G. Berman B. Bertola F. . (2025). Molecular alterations in semen of per-and polyfluoroalkyl substance exposed subjects: association between DNA integrity, antioxidant capacity and lipoperoxides. Antioxidants (Basel) 14, 792. doi: 10.3390/antiox14070792. PMID: 40722896 PMC12291827

[B74] Massardo S. Verzola D. Alberti S. Caboni C. Santostefano M. Eugenio Verrina E. . (2024). MicroRaman spectroscopy detects the presence of microplastics in human urine and kidney tissue. Environ. Int. 184, 108444. doi: 10.1016/j.envint.2024.108444. PMID: 38281449

[B75] Mathew A. S. Harjith A. Guruvayoorappan C. (2025). Assessing microplastics as a novel threat to maternal-fetal health: placental barrier penetration and fetal developmental consequences. Reprod. Toxicol. 137, 109021. doi: 10.1016/j.reprotox.2025.109021. PMID: 40750000

[B76] Maxwell D. L. Oluwayiose O. A. Houle E. Roth K. Nowak K. Sawant S. . (2024). Mixtures of per- and polyfluoroalkyl substances (PFAS) alter sperm methylation and long-term reprogramming of offspring liver and fat transcriptome. Environ. Int. 186, 108577. doi: 10.1016/j.envint.2024.108577. PMID: 38521043

[B77] Meng L. Tian H. Lv J. Wang Y. Jiang G. (2023). Influence of microplastics on the photodegradation of perfluorooctane sulfonamide (FOSA). J. Environ. Sci. (China) 127, 791–798. doi: 10.1016/j.jes.2022.07.004. PMID: 36522106

[B78] Meng X. Yin K. Zhang Y. Wang D. Lu H. Hou L. . (2022a). Polystyrene microplastics induced oxidative stress, inflammation and necroptosis via NF-κB and RIP1/RIP3/MLKL pathway in chicken kidney. Toxicology 478, 153296. doi: 10.1016/j.tox.2022.153296. PMID: 36029908

[B79] Meng X. Zhang J. Wang W. Gonzalez-Gil G. Vrouwenvelder J. S. Li Z. (2022b). Effects of nano- and microplastics on kidney: physicochemical properties, bioaccumulation, oxidative stress and immunoreaction. Chemosphere 288, 132631. doi: 10.1016/j.chemosphere.2021.132631. PMID: 34688716

[B80] Meppelink S. M. Kolpin D. W. LeFevre G. H. Cwiertny D. M. Givens C. E. Green L. A. . (2025). Assessing microplastics, per- and polyfluoroalkyl substances (PFAS), and other contaminants of global concern in wadable agricultural streams in Iowa. Environ. Sci. Process. Impacts 27, 1401–1422. doi: 10.1039/d4em00753k. PMID: 40227795 PMC12826579

[B81] Metcalfe C. D. Bayen S. Desrosiers M. Muñoz G. Sauvé S. Yargeau V. (2022). An introduction to the sources, fate, occurrence and effects of endocrine disrupting chemicals released into the environment. Environ. Res. 207, 112658. doi: 10.1016/j.envres.2021.112658. PMID: 34990614

[B82] Min K. Cuiffi J. D. Mathers R. T. (2020). Ranking environmental degradation trends of plastic marine debris based on physical properties and molecular structure. Nat. Commun. 11, 727. doi: 10.1038/s41467-020-14538-z. PMID: 32024839 PMC7002677

[B83] Montano L. Giorgini E. Notarstefano V. Notari T. Ricciardi M. Piscopo M. . (2023). Raman microspectroscopy evidence of microplastics in human semen. Sci. Total Environ. 901, 165922. doi: 10.1016/j.scitotenv.2023.165922. PMID: 37532047

[B84] Moriya H. Hisada R. Fujieda Y. Kono M. Amengual O. Kato M. . (2025). The increase of monocyte/high-density lipoprotein cholesterol ratio precedes recurrent thrombosis in patients with antiphospholipid syndrome. Rheumatol. (Oxford) 65. doi: 10.1093/rheumatology/keaf655. PMID: 41352985

[B85] Musella F. Guarnieri M. G. Amore S. Montano L. Bertola F. Micali S. . (2025). Molecular and computational studies reveal that per- and polyfluoroalkyl substances can impair protamine-DNA interaction, potentially inducing DNA damage. Biomolecules 15, 1279. doi: 10.3390/biom15091279. PMID: 41008586 PMC12467272

[B86] Mushuo Q. Tian Y. Li J. Qiu Y. Fan H. Hu Q. . (2026). Mitophagy and oxidative stress in chronic kidney disease (review). Mol. Med. Rep. 33, 61. doi: 10.3892/mmr.2025.13771. PMID: 41347814 PMC12710145

[B87] Mutalifu L. Xie W. Wang D. Zhi M. Guo Y. Wang J. (2025). Emerging per- and polyfluoroalkyl substance perfluoro-(3,5,7,9-tetraoxadecanoic) acid (PFO4DA) impairs steroidogenesis and spermatogenesis by suppressing StAR and CYP11A1 expression in mice. Ecotoxicol. Environ. Saf. 292, 117962. doi: 10.1016/j.ecoenv.2025.117962. PMID: 40009946

[B88] Navarathna C. M. Pray H. Rodrigo P. M. Arwenyo B. McNeely C. Reynolds H. . (2023). Microplastics and per- and polyfluoroalkyl substances (PFAS) analysis in sea turtles and bottlenose dolphins along Mississippi’s coast. Analytica 4, 12–26. doi: 10.3390/analytica4010003. PMID: 41725453

[B89] Nielsen F. Fischer F. C. Leth P. M. Grandjean P. (2024). Occurrence of major perfluorinated alkylate substances in human blood and target organs. Environ. Sci. Technol. 58, 143–149. doi: 10.1021/acs.est.3c06499. PMID: 38154793 PMC10785751

[B90] Niu Z. Duan Z. He W. Chen T. Tang H. Du S. . (2024). Kidney function decline mediates the adverse effects of per- and poly-fluoroalkyl substances (PFAS) on uric acid levels and hyperuricemia risk. J. Hazard. Mater. 471, 134312. doi: 10.1016/j.jhazmat.2024.134312. PMID: 38640681

[B91] O’Callaghan L. Olsen M. Tajouri L. Beaver D. Hudson C. Alghafri R. . (2025). Plastic induced urinary tract disease and dysfunction: a scoping review. J. Expo Sci. Environ. Epidemiol. 35, 770–784. doi: 10.1038/s41370-024-00709-3, PMID: 39217203 PMC12401723

[B92] Pan Y. Cui Q. Wang J. Sheng N. Jing J. Yao B. . (2019). Profiles of emerging and legacy per-/polyfluoroalkyl substances in matched serum and semen samples: New implications for human semen quality. Environ. Health Perspect. 127, 127005. doi: 10.1289/ehp4431. PMID: 31841032 PMC6957285

[B93] Passos R. S. Davenport A. Busquets R. Selden C. Silva L. B. Baptista J. S. . (2023). Microplastics and nanoplastics in haemodialysis waters: emerging threats to be in our radar. Environ. Toxicol. Pharmacol. 102, 104253. doi: 10.1016/j.etap.2023.104253. PMID: 37604358

[B94] Posnack N. G. (2014). The adverse cardiac effects of Di(2-ethylhexyl)phthalate and Bisphenol A. Cardiovasc. Toxicol. 14, 339–357. doi: 10.1007/s12012-014-9258-y. PMID: 24811950 PMC4213213

[B95] Prada A. F. Scott J. W. Green L. Hoellein T. J. (2024). Microplastics and per- and polyfluoroalkyl substances (PFAS) in landfill-wastewater treatment systems: a field study. Sci. Total Environ. 954, 176751. doi: 10.2139/ssrn.4901601. PMID: 39378946

[B96] Qian Y. Liu Z. Lv X. Cai X. Wei J. Zhang L. . (2025). PFOA exposure promotes prostate cancer progression by enhancing autophagy through m(6)A modification of MAPK15 mRNA. Ecotoxicol. Environ. Saf. 303, 118839. doi: 10.1016/j.ecoenv.2025.118839. PMID: 40784096

[B97] Qiu W. Ye J. Su Y. Zhang X. Pang X. Liao J. . (2023). Co-exposure to environmentally relevant concentrations of cadmium and polystyrene nanoplastics induced oxidative stress, ferroptosis and excessive mitophagy in mice kidney. Environ. pollut. 333, 121947. doi: 10.1016/j.envpol.2023.121947. PMID: 37270049

[B98] Qu H. Han Y. Wang C. Zheng D. Ni Y. Xiao X. (2025). Unveiling the research void: exploring the reproductive effects of PFAS compounds on male health. Adv. Exp. Med. Biol. 1469, 127–162. doi: 10.1007/978-3-031-82990-1_7. PMID: 40301256

[B99] Quan H. Ji F. Zhong Z. Li R. Lu H. (2025). PFAS in bottled water from China: high prevalence of ultrashort-chain compounds, health risks, and global insights. J. Hazard. Mater. 499, 140184. doi: 10.1016/j.jhazmat.2025.140184. PMID: 41138410

[B100] Ragusa A. Notarstefano V. Svelato A. Belloni A. Gioacchini G. Blondeel C. . (2022). Raman microspectroscopy detection and characterisation of microplastics in human breastmilk. Polymers (Basel) 14, 2700. doi: 10.3390/polym14132700. PMID: 35808745 PMC9269371

[B101] Rainieri S. Conlledo N. Larsen B. K. Granby K. Barranco A. (2018). Combined effects of microplastics and chemical contaminants on the organ toxicity of zebrafish (Danio rerio). Environ. Res. 162, 135–143. doi: 10.1016/j.envres.2017.12.019. PMID: 29306661

[B102] Rashid F. Ramakrishnan A. Fields C. Irudayaraj J. (2020). Acute PFOA exposure promotes epigenomic alterations in mouse kidney tissues. Toxicol. Rep. 7, 125–132. doi: 10.1016/j.toxrep.2019.12.010. PMID: 31938689 PMC6953769

[B103] Salawu O. A. Olivares C. I. Adeleye A. S. (2024). Adsorption of PFAS onto secondary microplastics: a mechanistic study. J. Hazard. Mater. 470, 134185. doi: 10.1016/j.jhazmat.2024.134185. PMID: 38579582

[B104] Sánchez-Resino E. Marquès M. Gutiérrez-Martín D. Restrepo-Montes E. Martínez M. Salas-Huetos A. . (2023). Exploring the occurrence of organic contaminants in human semen through an innovative LC-HRMS-based methodology suitable for target and nontarget analysis. Environ. Sci. Technol. 57, 19236–19252. doi: 10.1021/acs.est.3c04347. PMID: 37934628 PMC10722465

[B105] Sands M. Sachdeva A. Bukavina L. Irudayaraj J. (2025). Synergistic toxicity of PFAS and microplastic mixtures across five human cell lines. Environ. pollut. 386, 127256. doi: 10.1016/j.envpol.2025.127256. PMID: 41086909 PMC13050527

[B106] Sands M. Zhang X. Irudayaraj J. (2024). Kidney toxicology of a novel compound lithium bis(trifluoromethanesulfonyl)imide (LiTFSI, ie. HQ-115) used in energy applications: an epigenetic perspective. Sci. Total Environ. 955, 177019. doi: 10.1016/j.scitotenv.2024.177019. PMID: 39447891 PMC12186188

[B107] Schellenberger S. Liagkouridis I. Awad R. Khan S. Plassmann M. Peters G. . (2022). An outdoor aging study to investigate the release of per- and polyfluoroalkyl substances (PFAS) from functional textiles. Environ. Sci. Technol. 56, 3471–3479. doi: 10.1021/acs.est.1c06812. PMID: 35213128 PMC8928479

[B108] Shi L. Tao L. Zong Y. Gao C. Han B. Gao T. . (2025). Seminal per- and polyfluoroalkyl substance exposure and sperm quality impairment: from toxic target to rescue. Environ. Int. 200, 109533. doi: 10.1016/j.envint.2025.109533. PMID: 40409068

[B109] Soltanighias T. Umar A. Abdullahi M. Abdallah M. A. Orsini L. (2024). Combined toxicity of perfluoroalkyl substances and microplastics on the sentinel species Daphnia magna: implications for freshwater ecosystems. Environ. pollut. 363, 125133. doi: 10.1016/j.envpol.2024.125133. PMID: 39419463

[B110] Song S. Huang L. Zhou X. Han Y. Yu J. (2025). Impact of per- and polyfluoroalkyl substances on diabetic kidney disease. Front. Endocrinol. (Lausanne) 16. doi: 10.3389/fendo.2025.1594897. PMID: 40995593 PMC12454095

[B111] Stanifer J. W. Stapleton H. M. Souma T. Wittmer A. Zhao X. Boulware L. E. (2018). Perfluorinated chemicals as emerging environmental threats to kidney health: A scoping review. Clin. J. Am. Soc Nephrol. 13, 1479–1492. doi: 10.2215/cjn.04670418. PMID: 30213782 PMC6218824

[B112] Su W. An Z. Mei Y. Tan Z. Jiang Z. Zeng X. . (2025). Impact of per- and polyfluoroalkyl substances exposure on renal dysfunction: Integrating epidemiological evidence with mechanistic insights. Environ. pollut. 382, 126744. doi: 10.1016/j.envpol.2025.126744. PMID: 40588156

[B113] Sun Z. Wen Y. Wang B. Deng S. Zhang F. Fu Z. . (2023). Toxic effects of per- and polyfluoroalkyl substances on sperm: epidemiological and experimental evidence. Front. Endocrinol. (Lausanne) 14. doi: 10.3389/fendo.2023.1114463. PMID: 36891048 PMC9986484

[B114] Sunderland E. M. Hu X. C. Dassuncao C. Tokranov A. K. Wagner C. C. Allen J. G. (2019). A review of the pathways of human exposure to poly- and perfluoroalkyl substances (PFASs) and present understanding of health effects. J. Expo Sci. Environ. Epidemiol. 29, 131–147. doi: 10.1038/s41370-018-0094-1. PMID: 30470793 PMC6380916

[B115] Thompson R. C. Olsen Y. Mitchell R. P. Davis A. Rowland S. J. John A. W. . (2004). Lost at sea: where is all the plastic? Science 304, 838. doi: 10.1126/science.1094559. PMID: 15131299

[B116] Vega-Herrera A. Savva K. Lacoma P. Santos L. Hernández A. Marmelo I. . (2024). Bioaccumulation and dietary bioaccessibility of microplastics composition and cocontaminants in Mediterranean mussels. Chemosphere 363, 142934. doi: 10.2139/ssrn.4739125. PMID: 39053781

[B117] Vieira Y. Lima E. C. Foletto E. L. Dotto G. L. (2021). Microplastics physicochemical properties, specific adsorption modeling and their interaction with pharmaceuticals and other emerging contaminants. Sci. Total Environ. 753, 141981. doi: 10.1016/j.scitotenv.2020.141981. PMID: 32911167

[B118] Wang W. Guan J. Feng Y. Nie L. Xu Y. Xu H. . (2022). Polystyrene microplastics induced nephrotoxicity associated with oxidative stress, inflammation, and endoplasmic reticulum stress in juvenile rats. Front. Nutr. 9. doi: 10.3389/fnut.2022.1059660. PMID: 36687698 PMC9853403

[B119] Wang Y. L. Huang C. C. Zheng C. M. Liu W. C. Lee Y. H. Chiu H. W. (2024). Polystyrene microplastic-induced extracellular vesicles cause kidney-related effects in the crosstalk between tubular cells and fibroblasts. Ecotoxicol. Environ. Saf. 273, 116098. doi: 10.1016/j.ecoenv.2024.116098. PMID: 38368757

[B120] Wang Y. L. Lee Y. H. Hsu Y. H. Chiu I. J. Huang C. C. Huang C. C. . (2021). The kidney-related effects of polystyrene microplastics on human kidney proximal tubular epithelial cells HK-2 and male C57BL/6 mice. Environ. Health Perspect. 129, 57003. doi: 10.1289/ehp7612. PMID: 33956507 PMC8101928

[B121] Wang T. Li N. Zhao Z. Zhang Y. Lin L. Ding Y. . (2025). Microplastic contamination in kidney transplant donor tissues and its association with early post-transplant blood pressure changes in recipients: a multi-modal analytical study. J. Hazard. Mater. 497, 139728. doi: 10.1016/j.jhazmat.2025.139728. PMID: 40902535

[B122] Wang M. Liu Q. Zhang X. Jiang H. Zhang X. (2025). Identification and analysis of microplastics in human penile cancer tissues. Sci. Total Environ. 969, 178815. doi: 10.1016/j.scitotenv.2025.178815. PMID: 40015124

[B123] Wang T. Yang J. Han Y. Wāng Y. (2024). Unveiling the intricate connection between per- and polyfluoroalkyl substances and prostate hyperplasia. Sci. Total Environ. 932, 173085. doi: 10.1016/j.scitotenv.2024.173085. PMID: 38729377

[B124] Washburn R. L. Hibler T. Kaur G. Dufour J. M. (2022). Sertoli cell immune regulation: a double-edged sword. Front. Immunol. 13. doi: 10.3389/fimmu.2022.913502. PMID: 35757731 PMC9218077

[B125] Wei W. Huo H. Song X. Wang D. Huang H. Jiang F. (2025). Individual and mixed effects of PFAS on osteoporosis: insights from epidemiological and bioinformatic approaches. Toxicol. Lett. 413, 111731. doi: 10.1016/j.toxlet.2025.111731. PMID: 41022338

[B126] Wei Y. Zhou Y. Long C. Wu H. Hong Y. Fu Y. . (2021). Polystyrene microplastics disrupt the blood-testis barrier integrity through ROS-mediated imbalance of mTORC1 and mTORC2. Environ. pollut. 289, 117904. doi: 10.1016/j.envpol.2021.117904. PMID: 34371264

[B127] Wen Y. Rashid F. Fazal Z. Singh R. Spinella M. J. Irudayaraj J. (2022). Nephrotoxicity of perfluorooctane sulfonate (PFOS)-effect on transcription and epigenetic factors. Environ. Epigenet. 8, dvac010. doi: 10.1093/eep/dvac010. PMID: 35633893 PMC9134076

[B128] Wu Y. Lv Y. Ran S. Xie W. Zhou H. Zhang Z. . (2025). Gestational exposure to poly/perfluoroalkyl substances and risk of congenital structural malformations: a nested case-control study. Reprod. Toxicol. 138, 109074. doi: 10.1016/j.reprotox.2025.109074. PMID: 41015328

[B129] Xi J. Ji H. Li J. Li R. Xie Z. Jin L. . (2025). Associations of prenatal exposure to legacy and emerging PFAS with anogenital distance growth trajectories during early childhood: a prospective cohort study. J. Hazard. Mater. 495, 138999. doi: 10.1016/j.jhazmat.2025.138999. PMID: 40555014

[B130] Xiong X. Gao L. Chen C. Zhu K. Luo P. Li L. (2023). The microplastics exposure induce the kidney injury in mice revealed by RNA-seq. Ecotoxicol. Environ. Saf. 256, 114821. doi: 10.1016/j.ecoenv.2023.114821. PMID: 36989554

[B131] Xu W. Ye S. Liu W. Guo H. Zhang L. Wei S. . (2024). Single-cell RNA-seq analysis decodes the kidney microenvironment induced by polystyrene microplastics in mice receiving a high-fat diet. J. Nanobiotechnology 22, 13. doi: 10.1186/s12951-023-02266-7. PMID: 38167034 PMC10762848

[B132] Yakovenko N. Pérez-Serrano L. Segur T. Hagelskjaer O. Margenat H. Le Roux G. . (2025). Human exposure to PM10 microplastics in indoor air. PloS One 20, e0328011. doi: 10.1371/journal.pone.0328011. PMID: 40737229 PMC12310009

[B133] Yang H. Lai H. Huang J. Sun L. Mennigen J. A. Wang Q. . (2020). Polystyrene microplastics decrease F-53B bioaccumulation but induce inflammatory stress in larval zebrafish. Chemosphere 255, 127040. doi: 10.1016/j.chemosphere.2020.127040. PMID: 32416398

[B134] Yang W. Ling X. He S. Cui H. Wang L. Yang Z. . (2024). Perturbation of IP3R-dependent endoplasmic reticulum calcium homeostasis by PPARδ-activated metabolic stress leads to mouse spermatocyte apoptosis: A direct mechanism for perfluorooctane sulfonic acid-induced spermatogenic disorders. Environ. pollut. 343, 123167. doi: 10.1016/j.envpol.2023.123167. PMID: 38110051

[B135] Yashir N. Sun Q. Zhang X. Ma M. Wang D. Feng Y. . (2025). Co-occurrence of microplastics, PFASs, antibiotics, and antibiotic resistance genes in groundwater and their composite impacts on indigenous microbial communities: a field study. Sci. Total Environ. 961, 178373. doi: 10.1016/j.scitotenv.2025.178373. PMID: 39793130

[B136] Yu F. Wu J. Wang H. Bao Y. Xing H. Ye W. . (2024). Interaction of microplastics with perfluoroalkyl and polyfluoroalkyl substances in water: a review of the fate, mechanisms and toxicity. Sci. Total Environ. 948, 175000. doi: 10.1016/j.scitotenv.2024.175000. PMID: 39053539

[B137] Zeng X. W. Bloom M. S. Wei F. Liu L. Qin J. Xue L. . (2023). Perfluoroalkyl acids in follicular fluid and embryo quality during IVF: A prospective IVF cohort in China. Environ. Health Perspect. 131, 27002. doi: 10.1289/ehp10857. PMID: 36723383 PMC9891133

[B138] Zhang Q. Li S. Fang J. Hao Y. Lu J. Zhang L. . (2024). The combined effects of polystyrene of different sizes and cadmium in mouse kidney tissues. Ecotoxicol. Environ. Saf. 281, 116660. doi: 10.1016/j.ecoenv.2024.116660. PMID: 38944012

[B139] Zhang Y. Li Y. Gao N. Gong Y. Shi W. Wang X. (2023). Transcriptome and metabolome analyses reveal perfluorooctanoic acid-induced kidney injury by interfering with PPAR signaling pathway. Int. J. Mol. Sci. 24, 11503. doi: 10.3390/ijms241411503. PMID: 37511261 PMC10380573

[B140] Zhang X. Sands M. Lin M. Guelfo J. Irudayaraj J. (2024). *In vitro* toxicity of lithium bis(trifluoromethanesulfonyl)imide (LiTFSI) on human renal and hepatoma cells. Toxicol. Rep. 12, 280–288. doi: 10.1016/j.toxrep.2024.02.008, PMID: 38469334 PMC10925923

[B141] Zhang Y. Xu Y. Ding H. Yu W. Chen L. (2021). Prenatal exposure of female mice to perfluorononanoic acid delays pubertal activation of the reproductive endocrine axis through enhanced hepatic FGF21 production. Chemosphere 269, 128776. doi: 10.1016/j.chemosphere.2020.128776. PMID: 33131727

[B142] Zhang Q. Xu E. G. Li J. Chen Q. Ma L. Zeng E. Y. . (2020). A review of microplastics in table salt, drinking water, and air: direct human exposure. Environ. Sci. Technol. 54, 3740–3751. doi: 10.1021/acs.est.9b04535. PMID: 32119774

[B143] Zhao Q. Fang Z. Wang P. Qian Z. Yang Y. Ran L. . (2025). Polylactic acid micro/nanoplastic exposure induces male reproductive toxicity by disrupting spermatogenesis and mitochondrial dysfunction in mice. ACS Nano 19, 5589–5603. doi: 10.1021/acsnano.4c15112. PMID: 39869919

[B144] Zhao Q. Zhu L. Weng J. Jin Z. Cao Y. Jiang H. . (2023). Detection and characterization of microplastics in the human testis and semen. Sci. Total Environ. 877, 162713. doi: 10.1016/j.scitotenv.2023.162713. PMID: 36948312

[B145] Zheng P. Liu Y. An Q. Yang X. Yin S. Ma L. Q. . (2022). Prenatal and postnatal exposure to emerging and legacy per-/polyfluoroalkyl substances: Levels and transfer in maternal serum, cord serum, and breast milk. Sci. Total Environ. 812, 152446. doi: 10.1016/j.scitotenv.2021.152446. PMID: 34952085

[B146] Zhou P. Kong Y. Zhang D. Juhasz A. Zhang Q. Cui X. (2025). Influence of non-degradable and degradable microplastics on the bioavailability of per- and polyfluoroalkyl substance in mice: mechanism exploration. Sci. Total Environ. 977, 179381. doi: 10.1016/j.scitotenv.2025.179381. PMID: 40222253

[B147] Zhou X. Yan H. Hong Y. Ding Y. Chen J. Tang H. . (2025). PPARγ mediated lysosomal membrane permeabilization and lipophagy blockage were involved in microplastics and di (2-ethylhexyl) phthalate co-exposure induced immature testis injury. Free Radic. Biol. Med. 237, 615–630. doi: 10.1016/j.freeradbiomed.2025.06.023. PMID: 40532836

[B148] Zou H. Chen Y. Qu H. Sun J. Wang T. Ma Y. . (2022). Microplastics exacerbate cadmium-induced kidney injury by enhancing oxidative stress, autophagy, apoptosis, and fibrosis. Int. J. Mol. Sci. 23, 14411. doi: 10.3390/ijms232214411. PMID: 36430889 PMC9694236

